# Functional Relevance of Different Basal Ganglia Pathways Investigated in a Spiking Model with Reward Dependent Plasticity

**DOI:** 10.3389/fncir.2016.00053

**Published:** 2016-07-21

**Authors:** Pierre Berthet, Mikael Lindahl, Philip J. Tully, Jeanette Hellgren-Kotaleski, Anders Lansner

**Affiliations:** ^1^Numerical Analysis and Computer Science, Stockholm UniversityStockholm, Sweden; ^2^Department of Computational Biology, School of Computer Science and Communication, KTH Royal Institute of TechnologyStockholm, Sweden; ^3^Stockholm Brain Institute, Karolinska InstituteStockholm, Sweden; ^4^Institute for Adaptive and Neural Computation, School of Informatics, University of EdinburghEdinburgh, UK; ^5^Department of Neuroscience, Karolinska InstituteStockholm, Sweden

**Keywords:** basal ganglia, action selection, reinforcement learning, synaptic plasticity, dopamine, reward prediction error, Parkinson's disease

## Abstract

The brain enables animals to behaviorally adapt in order to survive in a complex and dynamic environment, but how reward-oriented behaviors are achieved and computed by its underlying neural circuitry is an open question. To address this concern, we have developed a spiking model of the basal ganglia (BG) that learns to dis-inhibit the action leading to a reward despite ongoing changes in the reward schedule. The architecture of the network features the two pathways commonly described in BG, the direct (denoted D1) and the indirect (denoted D2) pathway, as well as a loop involving striatum and the dopaminergic system. The activity of these dopaminergic neurons conveys the reward prediction error (RPE), which determines the magnitude of synaptic plasticity within the different pathways. All plastic connections implement a versatile four-factor learning rule derived from Bayesian inference that depends upon pre- and post-synaptic activity, receptor type, and dopamine level. Synaptic weight updates occur in the D1 or D2 pathways depending on the sign of the RPE, and an efference copy informs upstream nuclei about the action selected. We demonstrate successful performance of the system in a multiple-choice learning task with a transiently changing reward schedule. We simulate lesioning of the various pathways and show that a condition without the D2 pathway fares worse than one without D1. Additionally, we simulate the degeneration observed in Parkinson's disease (PD) by decreasing the number of dopaminergic neurons during learning. The results suggest that the D1 pathway impairment in PD might have been overlooked. Furthermore, an analysis of the alterations in the synaptic weights shows that using the absolute reward value instead of the RPE leads to a larger change in D1.

## Introduction

The BG have a parallel pathway structure suitable for conveying action commands, with both action promotion and suppression built in (DeLong, 1990; Graybiel, 1995, 2005; Houk et al., 1995; Mink, 1996; Redgrave et al., 1999). Originating with the medium spiny neurons (MSNs) of the striatum, two main pathways are distinguished by their expressed dopamine receptor type (i.e., D1 or D2). D1 and D2 receptors are mostly mutually exclusive and distributed equally throughout striatum (Gerfen, 1992). Specific stimulations of D1 or D2 pathways lead to an increase or decrease in motor response, respectively (Kravitz et al., 2010, 2012; Tai et al., 2012). Both types of MSNs receive similar afferent glutamatergic input from cortex, thalamus and the limbic system (McGeorge and Faull, 1989; Parent, 1990; Doig et al., 2010) and both pathways converge onto the output structures of the BG, the internal globus pallidus (GPi), and the substantia nigra pars reticula (SNr). It has been suggested that cortical, thalamic and limbic inputs inform BG about the current state of the environment. Furthermore, the striatum has been shown to encode action values and to serve as the interface between these inputs and the rest of the BG (Samejima et al., 2005). Neurons in the striatum also get diffuse dopaminergic inputs from the ventral tegmental area (VTA) and substantia nigra pars compacta (SNc; Parent, 1990). Phasic and tonic dopamine release is believed to be critical for synaptic plasticity, triggering bi-directional changes of the connections onto the two different types of MSNs in the striatum (Reynolds and Wickens, 2000; Surmeier et al., 2007; Berretta et al., 2008; Shen et al., 2008). This dopaminergic signal is commonly accepted as coding for the RPE, which is the difference between the expected and the actually received reward and resembles the temporal difference (TD) error in reinforcement learning algorithms (Berns et al., 2001; Suri and Schultz, 2001; Suri, 2002; Glimcher, 2011). Degeneration of dopaminergic neurons has been observed in patients with PD (Obeso et al., 2000) and is believed to cause impairment mainly in the indirect pathway (Kreitzer and Malenka, 2007; Kravitz et al., 2010).

It is commonly considered that the system should increase the weight to the relevant D1 population if an action led to an unexpected excess of reward, and to the D2 population if reward was less than expected. Meanwhile, the reward prediction (RP) system should also learn the value of the reward delivered. In the TD learning framework, a reinforcement only contributes to learning if it is not predictable (Sutton and Barto, 1998). Computational models based on the Actor-Critic framework and using TD learning have tried to reproduce the functional and architectural features of BG (for reviews: Gillies and Arbuthnott, 2000; Joel et al., 2002; Doya, 2007; Cohen and Frank, 2009; Samson et al., 2010; Schroll and Hamker, 2013). Additionally, most of the computational models of the BG have either focused on biological plausibility (Lindahl et al., 2013; Gurney et al., 2015) or functional reproduction of the behavior during learning or action selection (Limousin et al., 1995; Gurney et al., 2001; Frank, 2006; O'Reilly and Frank, 2006; Ito and Doya, 2009; Potjans et al., 2009; Stocco et al., 2010; Jitsev et al., 2012; Stewart et al., 2012; Collins and Frank, 2014). As a result, there has been limited focus directed toward implementing functional spike-based models, specifically those that can also simulate dopamine depletion (but see Potjans et al., 2011).

The central nervous system has been shown to be able to perform inference (Körding and Wolpert, 2004), and Bayesian probabilities can be represented by artificial neural networks and spiking neurons (Doya et al., 2007; Buesing et al., 2011; Boerlin et al., 2013). If the brain is representing information in a probabilistic manner, it is plausible that this is reflected on the level of neurons and synapses (Deneve, 2008; Tully et al., 2014). We have extended our previous computational model of BG based on a Bayesian Confidence Propagation Neural Network (BCPNN) learning rule derived from Bayesian inference (Berthet et al., 2012) with spiking neurons such that the plasticity probabilistically depends on the activity of neural populations, mimicking the RPE supposedly conveyed by dopaminergic neurons. This step enabled both the comparison with our previous more abstract implementation, as the general architecture was preserved, and also offered more biologically relevant predictions and analogy as the general architecture of the BG was better represented. The versatile framework of BCPNN has been implemented in the context of associative and working memory, memory consolidation, pattern completion and recognition, olfactory modeling, and data mining (Bate et al., 1998; Sandberg et al., 2000; Sandberg, 2003; Lundqvist et al., 2011; Meli and Lansner, 2013; Fiebig and Lansner, 2014; Kaplan and Lansner, 2014).

We evaluate the performance of the model in action selection and reinforcement learning tasks. The ambition here was to investigate how our previous top-down approach, enhanced with some more neurological details such as spiking neurons and volume transmission of dopamine, could offer insights, and predictions that could be biologically tested. We demonstrate that performance of the spiking model is similar to that of our previous abstract model. We further assess the impact of reducing dopaminergic neuron number during the simulation, mimicking PD, and exposing the roles of the D1 and D2 MSNs for the degraded performance.

## Model and methods

Extending a previous abstract model of the BG (Berthet et al., 2012), we implemented a spiking neuron model incorporating plasticity governed by spike-based BCPNN learning (Tully et al., 2014) that was globally modulated through volume transmission of dopamine (Potjans et al., 2010). Grounded in the hypothesis that the brain builds a model of the world by computing probabilities of occurrences and co-occurrences of events, BCPNN assumes synaptic weights and neuronal excitabilities are the substrate for storing these probabilities. It should be noted that not all components of the BG are included in the model as we instead abstracted them to their general functionalities (Delong et al., 1984; Mink, 1996; Bar-Gad et al., 2003; Romanelli et al., 2005; Sesack and Grace, 2010; Stephenson-Jones et al., 2013).

Our model was implemented in PyNEST (Gewaltig and Diesmann, 2007; Eppler et al., 2008) and simulations ran on a CRAY XC30 system. A simulation of the 725 neuron, 70,000 synapse network for 15 min biological time took around 120 min when executed on 20 cores. Details of the parameters and their values, as well as the source code, are provided as Supporting Information (Tables S1, S2).

### Competition between the D1 and D2 pathway for the selection

In the input layer of our network, different populations were assumed to code for the various states. These states conveyed contextual information about the environment and represented the cortical, thalamic and limbic inputs, referred to as “cortical” in the following, to the BG. In biology, the functional topology of BG implies that polysynaptic projections from D1 and D2 MSNs in the striatum code for the same action, and therefore inhibit or excite a population of neurons coding for that same action in GPi/SNr (Alexander et al., 1986; Nambu, 2008; Freeze et al., 2013). Thus, in our model, specific populations of neurons in the D1 and D2 pathways represented the different actions. We used a model with three states and three actions for all the simulations presented in this work. As reported in biology, MSNs in the striatum layer belong either to matrisomes or to striosomes (Gerfen, 1989; Johnston et al., 1990; Nakamura et al., 2009). Both types of MSNs receive similar afferent glutamatergic input from cortex, thalamus and the limbic system (McGeorge and Faull, 1989; Parent, 1990; Doig et al., 2010), and topographically organized projections from cortex and thalamus target both the matrix and striosomal compartments of the striatum (Joyce et al., 1986; Graybiel et al., 1987; Gerfen, 1992; Crittenden and Graybiel, 2011). It has also been shown that matrisomes are preferentially targeted by sensori-motor related neurons, whereas striosomes receive inputs mostly from the limbic system, orbito-frontal and pre-frontal cortex (Eblen and Graybiel, 1995; Graybiel, 2008; Crittenden and Graybiel, 2011). In our model, we interpreted the striosome/matrisome organization in the striatum as carrying a functional representation, similarly to its suggested actor-critic apparatus implementation (Houk et al., 1995). The MSNs in the matrisomes received connections from all neurons in the state coding input layer and these connections are modified using a dopamine dependent BCPNN plasticity rule. A matrisome was defined as a specific compartment of the matrix and coded for a specific action, whereas striosomes were instrumental to compute the expected reward for the state-action pairings. As suggested in our previous work (Berthet and Lansner, 2014), it was not only the neuronal activity of these populations but also the synaptic weights of their connections that coded for their relative values, i.e., action or state-action pairings.

Furthermore, in our model, each action was coded twice in the matrix, once for each of the D1 and D2 pathways. These D1 and D2 MSNs differed in the sign of the connections they sent to their respective action coding sub-population in the following layer, representing the GPi/SNr output layer of the BG. D1 neurons sent inhibitory projections and D2 neurons sent excitatory projections to the same specific action coding population in the subsequent layer. In the text we refer to inhibition and promotion of an action as D2 and D1, respectively. Therefore, we emphasize the perspective taken from the overall effect of activity on action selection in these pathways. Additionally, each action coding matrisomal population sent inhibitory connections to the other action coding populations sharing the same dopamine receptor type, i.e., D1 to other D1 and D2 to other D2 (Kemp and Powell, 1971; Taverna et al., 2008; Tepper et al., 2010; Szydlowski et al., 2013).

The motor circuit within the striato-pallidal system receives a continuous delayed read-out of cortical motor activity and issues an output directed through the thalamus mainly to pre-motor cortical regions (Marsden and Obeso, 1994; Kimura et al., 2004; McHaffie et al., 2005). A topographical organization has furthermore been reported on the thalamo-striatal connections (Mengual et al., 1999). It has thus been hypothesized that these feedback loops represent an efference copy that informs upstream populations, which otherwise would only get inputs about the state of the environment, of the eventually selected action (Redgrave and Gurney, 2006; Schroll and Hamker, 2013; Fee, 2014; Lisman, 2014). Based on this, the role of the efference copy in our model was to ensure that the current state-coding neurons fired simultaneously with the neurons coding for the selected action in the striatum, as suggested by Fee (2014) and Lisman (2014).

Neurons in GPi/SNr were driven to a baseline activity of around 35 Hz in the absence of matrisomal input. This is within the range of experimental data on GPi (Delong et al., 1984), but data for SNr suggest a broader dispersion (Gernert et al., 2004; Atherton and Bevan, 2005; Freeze et al., 2013).

### Reward prediction by the striosomes

Striosomes are widely distributed within the striatum. It has been reported that striosomes are specifically avoided by sensori-motor projections (Flaherty and Graybiell, 1993). They are also thought to be the only striatal neurons to project directly to the dopaminergic neurons in SNc (Lévesque and Parent, 2005). Thus, it has been suggested that they could convey reward predictions in a similar fashion as matrisomes code action values (Houk et al., 1995; Amemori et al., 2011; Morita et al., 2012; Stephenson-Jones et al., 2013). Even though synaptic plasticity has been reported at synapses on to dopaminergic neurons in VTA, there is not enough data, to our knowledge, to specify the properties of this plasticity (Bonci and Malenka, 1999; Jones et al., 2000; Lüscher and Malenka, 2011). In our model, we assumed that development and previous experience had segregated sub-populations into coding for exclusive state-action combinations. This suggests a representation of the different state-action pairings instead of distinct states and actions. The striosomal MSNs received connections from the state layer as well as the efference copy in such a way that only one specific state-action coding sub-population would get activated, more specifically the one receiving inputs from the currently active state and selected action (cf. Discussion for a comment on this particular setup). One aim of our model is thus to test the possible role of synaptic plasticity in the RP pathway from striosomes to dopaminergic neurons. Additionally, we artificially inserted a connection delay from the efference copy poisson generator to the striosome equal to the fixed duration of the efference copy phase in order to induce the activation of the relevant striosomal sub-population concomitantly with the delivery of the reward, and therefore the change of the external incoming activation given to the dopaminergic neurons. Hard coding the delay between the selection of the action and the reward delivery obviously did not allow for variable delays and was therefore less flexible than in biology. But we acknowledge that relaxing the constraint of delivering the reward at a fixed time would require a more complex temporal processing in the model, which lies outside the scope of this work, but could involve eligibility traces or spectral timing in the striosomes (Brown et al., 1999; Cardinal, 2006; Daw et al., 2006; Jin et al., 2009; Rivest et al., 2010; Morita et al., 2012; Gershman et al., 2014; Ruan et al., 2014).

### RPE modulates plasticity

It has been experimentally shown that dopaminergic neurons in SNc and VTA innervate both the striosome and matrix compartments (Gerfen et al., 1987; Joel and Weiner, 2000; Matsuda et al., 2009; Ilango et al., 2014) and that the release of dopamine serves as a non-specific reward signal that affects both compartments (Matsuda et al., 2009; Threlfell and Cragg, 2011). The extracellular dopamine concentration seems to be critical for modulating plasticity (Pawlak and Kerr, 2008; Pawlak et al., 2010), and its phasic levels are believed to code for the RPE (Schultz et al., 1997; Hollerman and Schultz, 1998; Bayer and Glimcher, 2005). Therefore, the dopamine feedback in our model was unspecific, i.e., it conveyed a global signal, representing the RPE and regulating the dopamine dependent synapses. However, in the RP pathway, specific reward value predictions were made for each state-action pairing (one for each state-action combination).

It should be noted that this model differs from TD algorithms as it does not bootstrap the estimated values of the next states or actions in order to use them in the update of the current value, but instead depends on the actual reward and its future reward-independent predicted value.

### Reward mapping

The reward mapping during simulated trials was consistent within the same block of 40 trials. A reward was delivered, i.e., the external excitatory input of the dopaminergic neurons was increased for a pre-defined period and then set back to baseline, if when in state *i*, the action selected *j* verified

(1) $$\begin{array}{l} \left(\left(i+b\right)mod\;a\right)\equiv j \end{array}$$

where *b* is the block number starting at zero, and *a* = 3 is the number of actions. The reward was delivered every time the correct action was selected. Therefore, if there was a change in the dopaminergic neurons firing rates, it meant that there was a mismatch between the inhibition sent from the active striosomes, i.e., the expected reward for this state-action pairing, and the external, reward mapping dependent excitation. The external reward delivery, or its absence, was coded by a change in the firing rate of the driving Poisson generator of the dopaminergic neurons: from baseline to high if a reward was obtained, or to low if no reward was obtained. Values of the static weights and delays, as well as those of the membrane voltage, threshold and reset value for the neuron model were all sampled from normal distributions.

### Phases of a trial

The whole simulation comprised several blocks, with each block comprising several trials. A trial consisted of four successive phases corresponding to four simulation times and lasted 1.5 seconds (**Figure 2A** details a single trial). At first during the selection phase, a state was defined by 30 Hz activation of a specific cortical population by Poisson inputs. The remaining populations of this layer also received Poisson inputs, but only at 3 Hz in order to simulate background noise. All the state coding populations had the same number of neurons (cf. Table S1 for a summary of the values used).

This activity flowed downstream through the D1 and D2 pathways to the GPi/SNr, but also to the striosomes. At the end of this phase, a softmax function was carried out on the spike counts of the three action coding populations in GPi/SNr in order to select an action (Daw and Doya, 2006). We first normalized the spike counts and then applied the softmax on the inverse distribution, as the goal was to have the action coded by the least active population be the one with the highest probability of being selected. Driving the GPi/SNr to fire in the absence of additional inputs allowed the D1 pathway to have an impact on selection by decreasing the spike count of the action coding population in GPi/SNr which was rewarded during the next occurrence.

For the second phase of the trial, the efference copy of the selected action was set to fire at a high rate while keeping the current state coding population active. This joint activity also enabled a single sub-population of a striosome to fire at around 15 Hz. The emitted spikes from this sub-population arrived at the dopaminergic neurons at the same time as the external reward mapping dependent excitation was applied (which happened in the following phase). Before the next phase of the trial, the reward had to be computed based on the current state, the selected action and the current block as described previously. The external input to the dopaminergic neurons was accordingly set to a higher (correct trial) or to a lower (error) value than baseline.

The third phase therefore represented the actual learning. Learning and plasticity occurred in the system at all dopamine dependent BCPNN synapses. For the fourth and last phase of the trial, the efference copy and the state layers were reset to their background noise and driven by low activity, and the dopaminergic neurons were set back to their baseline firing rate. This was done to avoid overlapping effects between trials (**Figure 2** displays an example of the activity in the network during 20 trials).

### Tests and lesioning of different pathways

We recorded the performance of the model as a moving average of success. A trial was correct when the selected action was the one leading to the reward. As defined previously (Equation 1), there was only one correct action for each state. The reward mapping was changed for each block of trials. Weight values were accessed every 250 ms of simulation. The mean weights and their standard deviations (SD) were computed based on 20 simulations for each condition. The success ratio was normalized so that chance level was 1/3, and the maximum score was 1.

We tested the impact of each pathway on the performance. To this end, we removed any contribution from a specific pathway on the selection: we set the outgoing weights of the corresponding striatal population, be it D1, D2, or RP, to zero. The population was consequently still active as it was receiving other inputs. However, its own output was blocked. For the condition without the RP pathway, only the absolute reward value impacted the dopaminergic neuron population. In order to assess the role of the efference copy, we ran simulations where it was removed. Similarly, we tested a condition where the lateral inhibition in striatum was removed. We also simulated the degeneration of dopaminergic neurons in SNc as observed in Parkinson's disease (Obeso et al., 2000). This was tested by silencing portions of these dopaminergic neurons (16, 33, and 66%), preventing them from having any further impact on the dynamics. This occurred after eight blocks of otherwise standard simulations. Performance was represented by the moving average of the success ratio in the successive three-way choice task, which quantified the learning capabilities of the model.

### Synaptic plasticity model

A two-factor learning rule, such as the standard spike timing dependent plasticity (STDP) (Bell et al., 1997; Markram et al., 1997; Bi and Poo, 1998), does not sufficiently characterize the dynamics of cortico-striatal plasticity (Pfister and Gerstner, 2006; Farries and Fairhall, 2007; Izhikevich, 2007; Legenstein et al., 2008; Frémaux et al., 2010, 2013; Paille et al., 2013; Gurney et al., 2015). Additionally to the pre- and post-synaptic spike timing, the dopamine level and MSN dopamine receptor type have been shown to be involved (Reynolds and Wickens, 2000; Surmeier et al., 2007; Berretta et al., 2008; Pawlak and Kerr, 2008; Shen et al., 2008; Yagishita et al., 2014). A recently described variation of STDP for cortico-striatal plasticity, implemented in a simple network with spiking neurons and derived from the plasticity observed in experiments, featured additional variables taking into account dopamine signaling and receptor types (Gurney et al., 2015). It offered dynamics comparable to our learning with one notable difference: the weight updates in a pathway, D1 or D2, was not restricted by the sign of the RPE. They did not however investigate the RP pathway and the computation of the RPE.

The spike-based BCPNN learning rule computes traces based on activity and co-activity in pre- *i* and post-synaptic *j* neurons. This is done in order to estimate the probability of the postsynaptic neuron being active given that the pre-synaptic one fires. The order of firing of the pre- and post-synaptic neurons is not critical here (unlike in Shen et al., 2008, but similar to Yagishita et al., 2014). The model takes advantage of the RPE as the learning rate, similar to Actor-Critic models (Suri and Schultz, 1999; Joel et al., 2002; Cohen and Frank, 2009) and reinforcement learning frameworks (Sutton and Barto, 1998). Specifically, three synaptic traces consisting of exponentially weighted moving averages are computed in order to estimate the probabilities of pre- and post-synaptic activation as well as their joint activations. The synaptic weight *w*_*ij*_ between the pre- and post-synaptic neurons can then be inferred from these traces. The RP pathway also exhibits dopamine dependent BCPNN plasticity to learn to predict the probability of reward given the current state and selected action.

The pre- *S*_*i*_ and post-synaptic *S*_*j*_ spike trains are defined by summed Dirac delta pulses with respective spike times $t_{sp}^{i}$ and $t_{sp}^{j}$:

(2) $$\begin{array}{l} S_{i}\left(t\right)=\sum_{sp}\delta \left(t-t_{sp}^{i}\right)\;S_{j}\left(t\right)=\sum_{sp}\delta \left(t-t_{sp}^{j}\right) \end{array}$$

Traces with the fastest dynamics, *Z*_*i*_ and *Z*_*j*_, are exponentially smoothed spike trains:

(3) $$\begin{array}{l} \tau_{Z_{i}}\frac{dZ_{i}}{dt}=\frac{S_{i}}{f_{\max\Delta t}}-Z_{i}+\varepsilon \;\tau_{Z_{j}}\frac{dZ_{j}}{dt}=\frac{S_{j}}{f_{\max\Delta t}}-Z_{j}+\varepsilon \end{array}$$

which lowpass filters pre- and post-synaptic activity with time constants τ_*Z*_*i*__ and τ_*Z*_*j*__, like what would be expected from rapid Ca^2+^ influx via NMDA channels or voltage-gated Ca^2+^ channels. It is assumed that each neuron could fire maximally at *f*_max_ Hz and minimally at ε*f*_max_ Hz, which represents absolute certainty and doubt regarding the evidential context of the input. Within that range, firing levels correspond to the estimated probability. Each spike event had a duration of Δ*t* ms.

These *Z* traces are then passed on to the *E* eligibility traces:

(4) $$\begin{array}{l} \tau_{e}\frac{dE_{i}}{dt}=Z_{i}\;-\;E_{i}\;\tau_{e}\frac{dE_{j}}{dt}=Z_{j}\;-\;E_{j}\;\tau_{e}\frac{dE_{ij}}{dt}=Z_{i}Z_{j}\;-\;E_{ij} \end{array}$$

where, in order to track the coincident activity from the *Z* traces, a separate equation is introduced. τ_*e*_ is the time constant for these traces which are assumed to represent intracellular Ca^2+^-dependent processes (Fukunaga et al., 1993). The *E* traces then are used in the computation of the *P* traces, whose longer time courses are inspired by processes like gene expression or protein synthesis. These values represent the final probability estimates based on smoothed activity levels:

(5) $$\begin{array}{l} \tau_{p}\frac{dP_{i}}{dt}=\kappa \left(E_{i}-P_{i}\right)\;\tau_{p}\frac{dP_{j}}{dt}=\kappa \left(E_{j}-P_{j}\right)\;\tau_{p}\frac{dP_{ij}}{dt}=\kappa \left(E_{ij}-P_{ij}\right) \end{array}$$

where κ is the RPE value and τ_*p*_ the time constant of these *P* traces.

In the absence of external inhibition from the striosomes, dopaminergic neurons were driven by an external Poisson process to a baseline activity of around 10 Hz, and, were set to fire at around 14 and 6 Hz for the delivery and non-delivery of the reward, respectively (Kiyatkin and Stein, 1995; Robinson et al., 2004; Ungless et al., 2004). At this baseline activity, the RPE was zero. When it deviated from baseline, the RPE became non-zero and enabled plasticity (Calabresi et al., 2000; Reynolds and Wickens, 2002; Wickens et al., 2003; Surmeier et al., 2007; Shen et al., 2008). Two cases could therefore occur: an increase of the firing rate of the dopaminergic neurons resulting in a positive RPE, or a decrease resulting in a negative RPE. These cases corresponded to biological processes (Hollerman and Schultz, 1998) shown to be sufficient for behavioral conditioning (Lavin et al., 2005; Tsai et al., 2009). D1 *P* traces are updated only if the RPE > 0 and D2 ones only if the RPE < 0 (Frank, 2005; Shen et al., 2008; Nair et al., 2015). The RPE value is computed from the spiking activity it receives from the dopaminergic neurons via the volume transmitter as follows:

(6) $$\begin{array}{l} RPE=\kappa ={\left(\sigma_{dopa}\left(\beta_{dopa}+q\right)\right)}^{\lambda } \end{array}$$

β_*dopa*_ is the value that biases the RPE to 0 when the dopaminergic neurons fire at baseline level. σ_*dopa*_ and λ are the gain and the power, respectively, and are different for matrisomes and striosomes. By using λ = 2 for the striosomes, κ is always positive and used to enable plasticity without respect to the sign of the RPE. However, λ = 7 was used for the matrisomes in order to retain information about the sign. The exponentiation helps to decrease the impact of small variations while increasing the impact of large variations. *q* was the filtered dopaminergic spike activity and acted as a proxy for the dopamine level in the model:

(7) $$\begin{array}{l} \tau_{q}\frac{dq}{dt}=\sum_{sp}\delta \left(t-t_{sp}^{dopa}\right)-q \end{array}$$

with τ_*q*_ as the time constant of volume transmission and $t_{sp}^{dopa}$ the spike times of the dopaminergic neurons.

Weights *w*_*ij*_ and biases β_*j*_ (cf. section Neuron Model) are computed from the final learning rule equation:

(8) $$\begin{array}{l} \beta_{j}=\log\left(P_{j}\right)\;w_{ij}=\log\left(\frac{P_{ij}}{P_{i}P_{j}}\right) \end{array}$$

The resulting synaptic learning rule was Hebbian and bidirectional, i.e., synapses show both LTD and LTP (Reynolds and Wickens, 2000; Shen et al., 2008; Pawlak et al., 2010; Yagishita et al., 2014). With the parameters used here, the precise order of firing of the pre- and post-synaptic neurons is not necessarily critical for the sign of the weight update, contrary to what is commonly using STDP learning rules (Gurney et al., 2015). The directionality of the update depended more on the correlated activity during a defined time interval (Tully et al., 2014).

Weights of a specific connection can grow alternatively positive or negative due to the logarithmic term in Equation (8). This could be understood as part of a microcircuit comprising a direct excitatory connection and a di-synaptic connection via an inhibitory interneuron, or as representing the axo-axonic ionotropic glutamate receptor-mediated excitation of the nerve of terminals of inhibitory neurons (Ren et al., 2007, but see Hull et al., 2009; Merchán-Pérez et al., 2009). A net positive weight means that the excitatory contribution would dominate over the inhibitory one and vice versa. In this work, we constrained the weights of the cortico-matrisomal connections to be only positive, setting a hard lower bound of 0 on *w*_*ij*_, but allowed the weights of the connections from striosomal neurons on to dopaminergic neurons to alternate between positive or negative values, as to not make a distinction based on the receptor types expressed in this pathway (cf. Discussion for some considerations). Consequently, the RP pathway cannot differentiate between the omission of an expected reward and an unexpected reward based only on the RPE. It is the degree of correlation between the pre- and post-synaptic activity that determines the increase and the decrease of the weights in RP, with the RPE acting only as a learning rate in this case.

The parameters of the learning rule in the RP pathway were set such that when pre-synaptic activity was associated with a high postsynaptic activity, e.g., after a reward has been obtained, the inhibitory weights would increase. This in turn would cause a decrease of the firing rate of the postsynaptic neurons during the next occurrence of that situation. Similarly, in the event of a reward omission or dip in the dopaminergic neuron firing rate, the weights from the active striosomal MSNs would decrease, possibly becoming negative, thereby driving up the activity in the postsynaptic population. This interplay between feed-forward inhibition/excitation, reward delivery, and the plasticity rule leads the striosomo-nigral weights to converge to a value where the dopaminergic neurons can fire at their baseline level, where the RPE is equal to zero.

### Neuron model

The neuron model implemented is of the leaky integrate-and-fire type with alpha function-shaped postsynaptic conductance (Meffin et al., 2004), which has been shown to be a useful model reduction of cortical neurons (Rauch et al., 2003). Parameters used are in the range of experimental observations (Pawlak and Kerr, 2008; Gittis et al., 2010). The neuron model is amended with *I*_β_*j*__, which accounts for the bias term β_*j*_ (Equation 8). The bias represents the prior probability of activation of a specific postsynaptic neuron. It enters the sub-threshold voltage *V*_*m*_ equation of the postsynaptic neuron according to:

(9) $$\begin{array}{lll} -C_{m}\frac{dV_{m}}{dt} & = & g_{L}\left(V_{m}-E_{L}\right)+\sum_{i=1}^{n}g_{ex,i}\left(V_{m}-E_{ex,i}\right)\\ +\;\sum_{i=1}^{n}g_{inh,i}\left(V_{m}-E_{inh,i}\right)+\phi I_{\beta_{j}} \end{array}$$

When threshold *V*_*th*_ is reached (*V*_*m*_ ≥ *V*_*th*_) a spike is generated and *V*_*m*_ is reset to the potential *V*_*res*_ for *t*_*ref*_ ms, representing the absolute refractory period. The total current flow across the membrane is determined by the membrane capacitance *C*_*m*_, the leak reversal potential *E*_*L*_, excitatory *E*_*ex*_ and inhibitory *E*_*inh*_ reversal potentials, the leak conductance *g*_*L*_, excitatory *g*_*ex*_ and inhibitory *g*_*inh*_ synaptic conductances, and *I*_β_*j*__ that is scaled by ϕ to represent an activity-dependent, intrinsic, hyperpolarizing current quantity. This could relate to the opening of some K^+^ channels. Postsynaptic conductances *g*_*ex*_ and *g*_*inh*_ are modified by the occurrence of an excitatory or inhibitory input event from one of the *n* presynaptic neurons at time $t_{sp}^{i}$ by:

(10) $$\begin{array}{l} g_{ex|inh,i}\left(t\right)=g_{max}w_{ij}\frac{t\;-\;t_{sp}^{i}\;-\;d}{\tau_{ex|inh}}e^{1-\frac{\left(t-t_{sp}^{i}-d\right)}{\tau_{ex|inh}}} \end{array}$$

This enables *g*_*ex*_ or *g*_*inh*_ to rise with finite duration τ_*ex*_ or τ_*inh*_ to its peak conductance *g*_*max*_*w*_*ij*_ at time $t-t_{sp}^{i}-d$ = τ_*ex*_ or τ_*inh*_, where *d* is the transmission delay, and to decay with time constant τ_*ex*_ or τ_*inh*_ thereafter.

## Results

**Figure 1:**
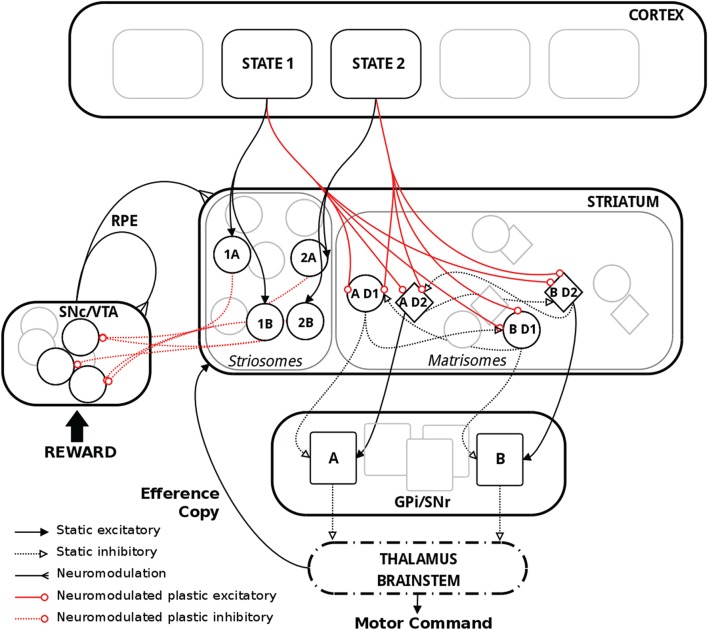
**Schematic representation of the model with relevant biological substrate**. In striatum, two actions A and B are here used as an example. The population “AD1” thus corresponds to the population of MSNs in the D1 pathway coding for action A. Striosomes and matrisomes are segregated for visual guidance, but they were intermingled in the model, with striosomes often referred to as “islands” in the matrix. Thalamus and brainstem were not explicitly implemented in the model but are shown above for completeness.

Our model consisted of the three main pathways in BG: D1, D2, and the dopaminergic RP feedback pathway (Figure 1). A state layer provided inputs to the striatum, which symbolized the set of possible actions. The striatum was divided into striosomes and matrisomes. Matrisomes consisted of D1 and D2 MSNs projecting to the output layer GPi/SNr, with inhibitory and excitatory projections representing the direct and indirect pathways, respectively. This simplified the polysynaptic circuit by providing functional dis-inhibition in the D1 pathway and dis-inhibition of excitatory neurons in thalamus or brain stem in the D2 pathway (Gerfen et al., 1990; Parent and Hazrati, 1995). Additionally, an efference copy informed the striatum about the action selected, which was based on the activity in GPi/SNr. The striosomes were part of the RP pathway and projected to dopaminergic neurons. The level of dopamine coded for the RPE and modulated plasticity in the system, which occurred at cortico-striatal synapses as well as synapses from striosomes targeting dopaminergic neurons. Learning in our model was dependent on four factors: pre- and post-synaptic activity, dopamine level and receptor type. Positive RPE triggered synaptic plasticity in D1, negative RPE triggered synaptic plasticity in D2, and either positive or negative RPE triggered synaptic plasticity in the RP pathway. The model comprised spiking integrate-and-fire model neurons and synaptic plasticity was based on the BCPNN learning rule.

### Learning the state-action mapping

**Figure 2:**
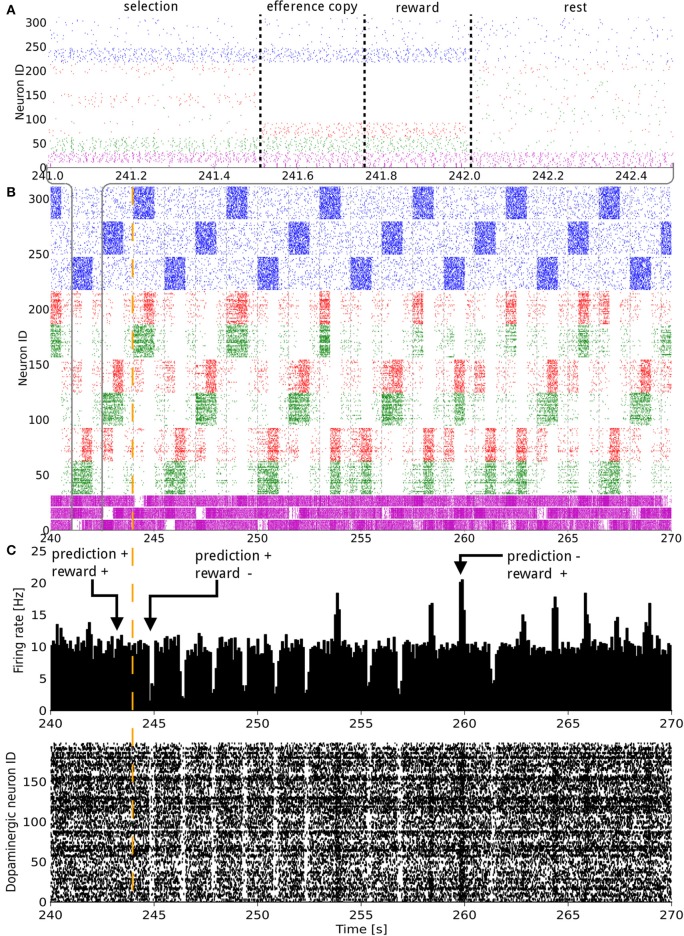
**Raster plot of the model consisting of 3 states and 3 actions, illustrated over 30 s of activity during a change in the reward mapping**. In **(A)**, a single trial is detailed. In **(A,B)** the states (blue), D1 (green), D2 (red), and GPi/SNr (purple) populations, grouped by representation coding, are shown. The indices of the states and actions begin from the top. For example, neurons with an ID between 160 and 220 represent the D1 and D2 populations coding for action 1. In **(C)** both the raster plot and a histogram of the dopaminergic neurons spiking activity are displayed. The width of the bins is 10 ms. A trial lasts for 1500 ms and starts with the onset of a new state. The simultaneous higher phasic firing rates in the D1 and D2 populations correspond to the population coding for the selected action receiving inputs form the efference copy. The vertical orange dashed line signals a change in the reward mapping.

The synaptic weights of the D1 and D2 MSNs for separate actions were successfully controlled by the RPE. Figure 2 shows the spiking activity of the cortical, striatal, pallido-nigral, and dopaminergic neurons during a change of reward mapping. The first trial of the new block was the one where the selection was incorrect (Figure 2: the trial immediately after the vertical orange dashed line), resulting in a dip in dopaminergic neuron activity starting at time = 244.75 s. This dip coded for a large negative RPE, which affected the D2 and RP pathways.

**Figure 3:**
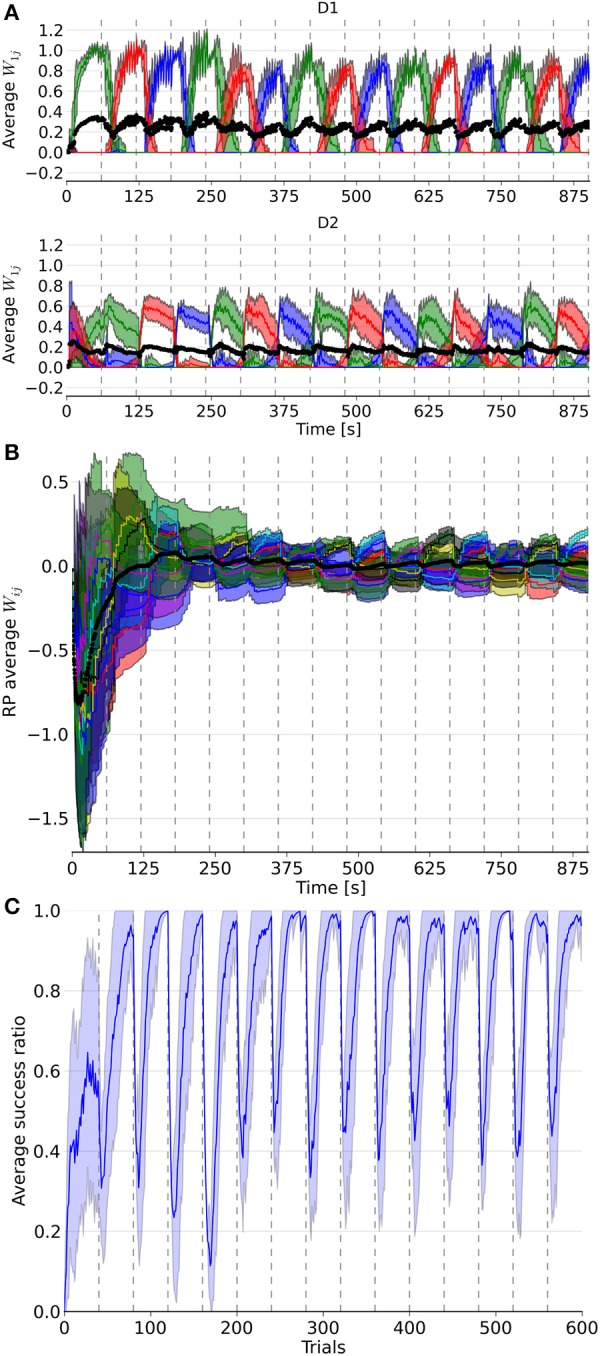
**Evolution of weights in the D1, D2, and RP pathways, as well as the performance over a simulation of 15 blocks with 40 trials each**. In **(A)**, the three colored lines represent the average weight of the three action coding populations in D1 and D2 from state 1. **(B)** represents the color coded average weights from the nine state-action pairing striosomal sub-population to the dopaminergic population. **(C)** displays the moving average success ratio of the model over the simulation. Vertical dashed gray lines denote the start of a new block. In **(A,B)**, the black line is the total average of the plotted weights. Color-coded shaded areas represent standard deviations.

The model was able to learn the correct reward mapping in simulations consisting of 15 blocks of 40 trials (Figure 3). The average success was well-above chance level and approached the maximum value 1 at the end of each block. This is comparable to the results from the abstract model, which featured 10 states and five actions on blocks of 200 trials (Berthet et al., 2012).

At the next occurrence of that same state, three trials later, the D2 population for the previously selected action fired more initially than during the previous occurrence of that state. This was, however, not sufficient to prevent the same action being selected. A decrease in the contrast of the firing rate of the three populations in GPi/SNr can be noticed for this trial. At the next occurrence of this state, the inhibition from D2 was enough this time to prevent the selection of that action. Fortunately, the selected action out of the two remaining possible was the rewarded one. This triggered a burst in the dopaminergic neurons activity and enabled plasticity in the D1 and RP pathways. The dopamine burst was noticeably bigger for each of the first-time rewarded state-action pairings of this new block, compared both to the subsequent successful selections and to the last correct trial of the previous block (Figure 2C).

Activity in a specific action coding D1 or D2 MSNs population produced a decrease or an increase in the firing rate of the corresponding action coding population in GPi/SNr, respectively. The activity triggered in matrisomes by the efference copy did not affect the selection, as at that point in time, the selection of the action has already been decided. However, it still mildly impacted the firing of the GPi/SNr neurons. The action selected was most likely to be the one with the lowest firing rate of the action coding populations in GPi/SNr as a result of the softmax.

It took several trials for the RP weights to settle into a stationary mode, in contrast to D1 and D2 weights (Figure 3). This was due to the discrepancy between the initial *P* trace values and the actual distribution of activity. Thus, for all the following statistical analyses and comparisons, we focused on the dynamics after the first four blocks.

Next, we considered the connection weights from the striosomes to SNc (Figure 3). The activity of the dopaminergic neurons was modulated by striosomal input. The delivery of a reward predicted by the RP pathway triggered little or no change in the firing rate of the dopaminergic neurons. The inhibition received from the striosomal sub-population coding for the relevant state-action pair compensated for the increased excitatory input brought by the reward on the dopaminergic neurons. However, the absence of delivery of an expected reward resulted in a large dip in activity, as the decrease of excitatory drive to SNc was added to the inhibition from the active striosomal MSNs. Conversely, the delivery of an unexpected reward provoked a burst in the dopaminergic neurons activity (Figure 2C). This burst was larger if the RP pathway had learned to expect the low activity in SNc, by having failed to obtain a reward for that specific state-action pair in the recent history.

During phasic dopamine changes, synaptic modification occurred not only between the active pre- and post-synaptic populations, but also at synapses where either only the pre- or post-synaptic population was active. The relative changes in magnitude taking place between inactive units were very small. Furthermore, changes in the weights were of opposite signs for the connections between co-active neurons and connections where only one end was active. These features led to some degree of homeostasis of the average weight (Figure 3).

Extinction and learning of a new reward mapping were the result of both a sharp increase of suppression from the then incorrect action coding D2 MSNs combined with a subsequent decrease of promotion by the D1 population coding for that action. The latter situation happened only once another action had been associated with the same state. Similarly, a decrease in the D2 weights of a population coding for an action happened when another action coding population saw its D2 weights increased, i.e., when this selected action was followed by a dip in the firing of the dopaminergic neuron. D2 weights showed the highest changes at the start of a block but then slowly decayed, in contrast to the D1 weights. That resulted from the larger RPE at the beginning of a new block, which was caused by the large difference between the expected reward associated during the previous block and the new negative outcome in the current block. Thus, the negative RPE impacted the D2 pathway initially, which helped the system to halt selection of the associated action thereafter. Eventually, inhibition became sufficient to enable the selection of another action, potentially triggering D1 plasticity in the case of a good choice. This matches results from the abstract version of the model along with experimental data (Groman et al., 2011).

Another observed phenomenon was that the dynamics of the weight changes were modified in D1 but not in D2 synapses. The rate of change in D1 got smaller after the initial updates within a block, whereas it stayed relatively constant in D2. As the RP pathway learned to correctly predict the reward, both the RPE and amplitude of the weight change decreased.

The slow decay of the D2 weights after the initial surge at the beginning of a block resulted from the small variations around baseline of the dopamine level. Note that it also affected D1 weights, as they gradually increased until the end of a block.

### Dopaminergic neuron loss

**Figure 4:**
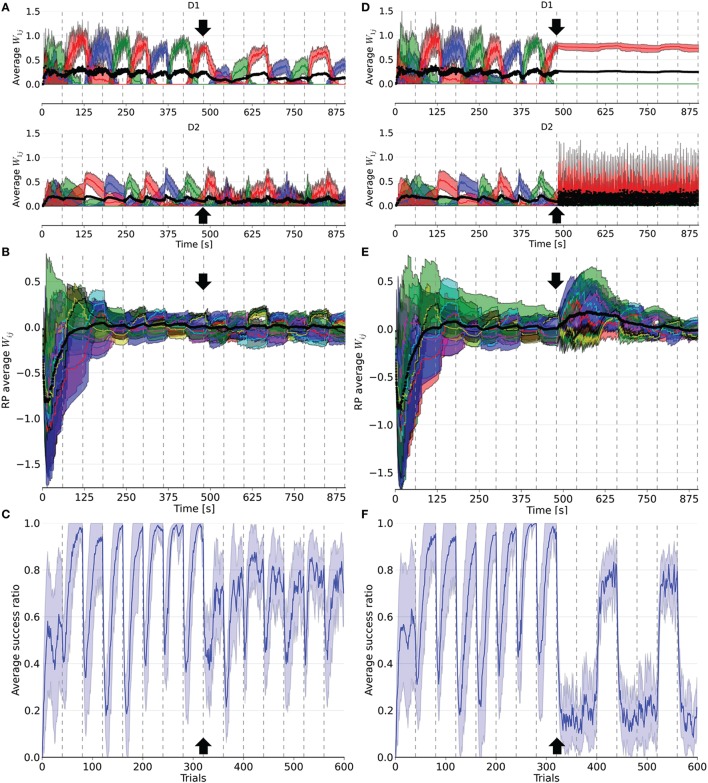
**Evolution of weights in the D1, D2, and RP pathways, as well as the performance over a simulation of 15 blocks of 40 trials each**. After eight blocks (black arrow), 16% (left column) or 66% (right column) of the dopaminergic neurons were rendered useless until the end of the simulation. In **(A,D)**, the weights represented are the average weight to each action coding population in D1 and D2 from state 1. **(B)** and **(E)** correspond to the average weights for each state-action pairing striosomal sub-population to the dopaminergic neuron population. **(C,F)** display the moving average success ratio of the model over the simulation. Vertical dashed gray lines denote the start of a new block. In **(A,B,D,E)**, the black line is the total average of the plotted weights. Color-code follows the one in Figure 3 and shaded areas represent standard deviations.

Figure 4 depicts the results from the PD simulations in which either 16% (PD16) or 66% (PD66) of the dopamine neurons were deleted. We also ran a test with a 33% decrease, which showed intermediate results (PD33, not shown). Deleting these neurons meant we removed both their incoming and outgoing connections, which silenced them and removed their effects on network activity. These deletions occurred at the end of the 8th block of the simulation. As a result, the tonic dopamine level settled to a value below the previous baseline, which the RPE was based on. Therefore, the RPE was negative by default, even outside of the reward delivery window.

**Figure 5:**
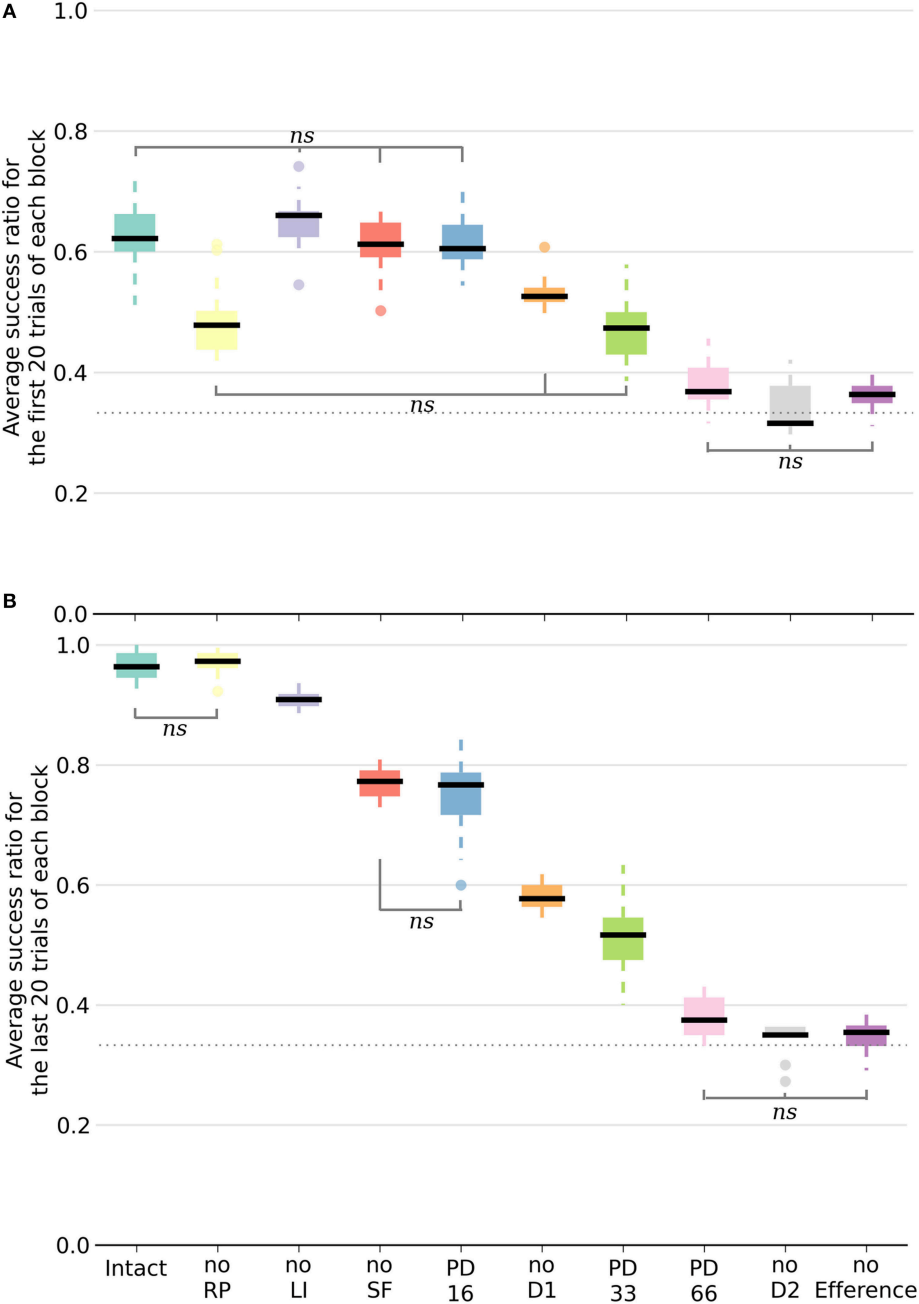
**Box plot of the mean success ratio and standard deviation of the examined conditions**. In **(A)**, the first 20 trials of each block were used whereas in **(B)**, the analysis was carried out on the last 20 trials. Data from the last seven blocks of the PD conditions were used. All differences are significant (*p* < 0.0001) unless stated otherwise (*ns, non-significant*). The horizontal dotted line represents chance level. For all conditions except PD33, PD66, noD2, and no Efference, differences within conditions between the first and last 20 trials are significant. NoSF stands for the condition without spontaneous firing of the GPi/SNr output nuclei and noLI for the condition without lateral inhibition in striatum. PD16, PD33, and PD66 display the results of the seven blocks following the deletion of, respectively, 16, 33, and 66% of the dopaminergic neurons.

Following deletion, performance immediately deteriorated for all conditions. The performance of PD33 and PD66 stabilized well-below the level indicated by the condition featuring only the D2 pathway (Figure 5) (PD33 mean 0.513 ± 0.061; *p* < 0.0001, PD66 mean 0.379 ± 0.035; *p* < 0.0001, comparisons based on the last 20 trials of the last seven blocks only). PD16 showed a relatively limited degradation in the success ratio (PD16 mean 0.749 ± 0.060).

The weights were also impacted by the dopamine decrease. In D1, they were updated less frequently and with less amplitude following the neuronal loss. Conversely, the negatively shifted RPE led the D2 weights to be updated more frequently and with larger amplitude. The extent of this asymmetry between D1 and D2 varied with the proportion of deleted dopaminergic neurons. For example, in PD66 the D2 weights of the action associated with the red color were more prominent in order to counterbalance the inadequate contribution of the D1 pathway in two out of three blocks. For PD16, however, both the D1 and D2 weights of the other two actions were diminished compared to their values before the PD simulation.

Interestingly, performance was rescued in blocks where the correct action was the last rewarded before the onset of PD once the number of dopaminergic neurons was decreased. This effect was more notable in the PD66 conditions (Figure 4F). Indeed, the contribution from D1 was relevant for the selection only once every three blocks, which depended upon the reward mapping. But even in this block, the level of success (mean 0.717 ± 0.041) was still well-below the one achieved before the simulated depletion of dopaminergic neurons. This resulted from the RPE not being able to reach a positive value even for correct selections, therefore inadequately triggering plasticity in D2. This ultimately caused D2 to hinder the selection of the action correctly promoted by D1.

However, in the two other blocks the contribution form D1 was erroneous. In such cases, the D2 pathway experienced a weight increase to that action in order to cancel the effect of D1. Once the system was able to select a different action than the one promoted by the D1 pathway, one action remained to be inhibited. As the D2 weights of the remaining incorrect action increased, those of the action promoted by D1 decreased, enabling this action to be incorrectly selected again.

The RP pathway was not sensitive to the sign of the RPE. Thus, weight updates in this pathway also became more frequent. As the dynamics of the remaining dopaminergic neurons were unaffected, the remaining weights between RP and SNc recovered to their values prior to neuronal loss after a few blocks of adaptation (Figures 4B,E). This is explained by the loss of connections to these neurons during deletion, meaning the remaining connections were those to the unaffected dopaminergic neurons.

We also tested the impact that the onset of PD had on the performance within a block. We doubled the number of trials in a specific block and decreased the number of dopaminergic neurons once reaching half the trials in that block. As this decrease was interpreted by the system as negative RPE, the subsequent trials exhibited some errors. However, the system's ability to revert to the correct action selection depended on the extent of the dopaminergic neuron decrease, with larger neuron deletions causing larger difficulties in the recovery. Additionally, the performance of the model benefited from an increase in the number of trials per block for the subsequent blocks, but only in PD16 and PD33, that is when the RPE could still become positive.

Overall, these simulations show that under PD conditions, the model faced great difficulties in learning new reward mappings. It succeeded in tasks that had already been learned when the contribution from the D1 pathway to the selection was adequate given the reward mapping.

### Functional relevance of different pathways

Inactivating a pathway proved to be a significant performance handicap, as all the conditions performed worse than the intact model with the exception of the noRP condition. Multiple comparisons were run with a one-way ANOVA. Figure 5 exhibits a representation of means, standard deviations and significance of the various conditions. In order to better capture the differing functional involvements during the learning process, we split the analysis in two based on the stage of the learning. We assumed that there would be an evolution between the initial transition phase relying mainly on the D2 pathway (first 20 trials of a block of 40 trials), and the later phase (last 20 trials of a block) where the D1 pathway would be critical in consistently singling out the appropriate action. Considering the different conditions, the success ratio was notably more severely affected without the D2 pathway (noD2) than without the D1 pathway (noD1). Both the former condition and the condition without the efference copy (noEfference) performed at chance level for the first and last stages of the block. This indicates that the efference copy was critical for this model.

This was also the case for the D2 pathway, but the selection pattern revealed that the system got stuck constantly selecting the same action: the one that was initially associated with the reward. Therefore, without the D2 pathway, and because of the absence of plasticity in D1 when the RPE is negative, the system did not change its selection and showed extreme perseveration. This critical role for the D2 pathway in changing the selection was also shown experimentally, where mice with D2 receptor knock-out (D2R-KO) performed significantly worse both in trial duration and success ratio compared to D1R-KO mice in a dynamic and uncertain environment (Kwak et al., 2014). In the model, the D1 weights were still updated every three blocks when the action selected was again the correct one. In the other two blocks, the RP pathway learned to expect a negative reward similar to the dynamics previously described for the intact model.

All of the conditions improved between the first and second halves of a block except the PD33, PD66, noD2 and noEfference ones. The removal of RP (noRP) resulted in a slower learning of the correct action after a change of reward mapping, as seen in the lower success ratio over the first 20 block trials. However, it eventually ended with the same success ratio average as the intact model over the last 20 trials. This underscores the beneficial role of the RP pathway in the early stages of a new acquisition. Even if the conditions without spontaneous firing of GPi/SNr (noSF), or without lateral inhibition between matrisomes (noLI) or noD1 improved during the second half of a block, they did not reach the same level as the intact and noRP conditions, which emphasizes a functional disorder not restricted to a specific learning stage. This held especially for the PD66 condition, as it was the one with the most limited improvements. In order to discard the slower learning capabilities of the model in these conditions, we tested them with extended lengths of blocks of 40 to 80 trials per block, and did not notice any improvement over subsequent trials, except for the PD33 condition (PD33 average success of the last 20 trials of a 40 trials block: 0.51 ± 0.06; last 20 trials of a 80 trials block: 0.64 ± 0.07).

Of noteworthy importance is that the PD33 and the noD1 conditions had relatively similar performances over the first half (i.e., the transition phase) of the 40 trials blocks. The noD1 improved, whereas the PD33 condition, which seemed to functionally rely only on the D2 pathway, did not. Even more dysfunctional was the PD66 condition, seemingly stuck to a low success ratio (0.379 ± 0.046). The results of the PD16 and of the noSF conditions were similar in that they both failed to improve as much as the intact condition, even though they had equivalent levels of performance over the first 20 trials of a block. The common feature of PD16 and of noSF was the reduced impact of D1 on the selection, through a reduced plasticity on D1 MSNs for the former and due to a direct lack of representation of the D1 inputs in the selection for the latter.

Surprisingly, the condition without lateral inhibition also performed almost as well as the intact model, showing quick transitions following a change in the reward mapping. However, the absence of the lateral inhibition provoked an increase in the baseline firing rate of the MSNs in the matrisomes, to around 15 Hz instead of < 1 Hz in the intact condition.

The condition without the RP pathway constrained the model so that an absolute reward had to be used instead of the RPE. Thus, the dopamine level changes depended only on the obtained reward. This was also a secondary effect of removing the efference copy. As the striosomal RP coding MSNs require simultaneous inputs from the relevant cortical neurons and efference copy to be active, the ablation of the latter rendered them silent. Therefore, in the noEfference condition, the model had to do without the efference copy and the RPE as well. The latter condition fared significantly worse than the condition without the RP pathway (*p* < 0.0001), and performed at chance level.

Returning to the noRP condition, we examined another metric to better comprehend the distinction with the intact model. We compared the average absolute amplitude change of the weights between the two conditions by measuring synaptic modifications. The average weight change between two consecutive trials was reduced in D1 and increased in D2 when the RP pathway was used (D1: mean 1.583 ± 1.662; D2: mean 0.861 ± 1.159) compared to the condition when it wasn't used but was instead based on the absolute reward value (D1: mean 3.535 ± 4.411, *p* < 0.0001; D2: mean 0.762 ± 1.074; *p* < 0.05). Based only on the reward value, cortico-striatal connection strengths onto D1 MSNs increased every time a response was correct, thus making the weights grow larger even though the correct mapping has been learned. As the RPE decreased because the RP pathway improved its predictions, the amplitude of the weight change was reduced. However, for D2, the amplitude was larger because the system expected the reward, and therefore the discrepancy was bigger when the outcome was negative e.g., at the beginning of a new block. Moreover, as this occurred only at the beginning of each block until the correct action was selected (and therefore the plasticity switched on to D1), the average change was larger than under the condition without RP, where the amplitude remained relatively constant.

Interestingly, the better results of the condition without spontaneous firing of GPi/SNr over the one without the D1 pathway imply some usefulness of the D1 pathway in the former condition. This can only happen in situations where the D2 pathway simultaneously tries to suppress the same action, i.e., disinhibiting the associated GPi/SNr population, thereby enabling inhibition from the D1 pathway to have a noticeable effect on the selection.

## Discussion

The presented model combines spiking neurons and biological data with a probabilistic learning rule. It uses reinforcement learning to select the correct action given a state and the associated expected reward value, implementing an efference copy mechanism as the critical way to control the localization of plastic changes. The dynamics and performance in a multiple choice task were quite similar to those achieved in a previous abstract model (Berthet et al., 2012). Furthermore, the activity of the model neurons coding the RPE during the various phases of learning, and the underlying mechanisms were congruent with theoretical and biological data on reinforcement learning, dopaminergic neuron activity and RPE (Schultz et al., 1997; Sutton and Barto, 1998; Pagnoni et al., 2002; Suri, 2002; Samejima et al., 2005; Groman et al., 2011). Dopaminergic neurons show a burst of activity for unexpected rewards, whereas the reward does not lead to any burst once fully expected. A dip in the dopaminergic neuron activity occurs when an expected reward is not obtained. These deviations from baseline control the plastic changes in the system.

The firing rates observed in the model, in populations with plastic synapses, are comparable with experimental data. In striatum, D1 and D2 MSNs' firing rate ranged from almost silent to around 30 Hz (Samejima et al., 2005; Kravitz et al., 2010). Additionally, the activity in both the D1 and D2 pathways increased during the selection phase due to co-activation of the two populations of MSNs, as reported in mice experiments (Cui et al., 2013; Tecuapetla et al., 2014) and computational models (Gurney et al., 2015). During the efference copy, only one action coding population was activated significantly while the other matrisomes were silent. With the inputs from the striosomes, the range of the firing rate of the dopaminergic neurons was extended to 1–18 Hz (Robinson et al., 2004; Bayer and Glimcher, 2005). Impacted by the activity in matrisomes, GPi/SNr neurons had a firing rate ranging from 0 to 80 Hz.

We have also lesioned the different pathways of the model. Compared to results from our previous abstract model, it might be surprising that the noD1 and noD2 conditions gave opposite rankings. In the work presented here, the best performance out of the two was obtained for the noD1 condition, whereas the noD2 condition resulted in a slightly superior success ratio in the abstract model. The learning rule was the same in both versions of the model. However, in the abstract model, the weights were updated in all pathways without restriction from the sign of the RPE. This meant that a negative RPE would decrease the cortico-striatal weights from the active state to the D1 population coding for the unrewarded selected action. This enabled the D1 pathway to unlearn an incorrect association without having to first rely on the relevant D2 suppression. This allowed the noD2 condition to perform relatively well, and even better than the condition without the D1 pathway (Berthet et al., 2012).

Using the RPE instead of the reward value improved the stability of the model without sacrificing plasticity, and would furthermore enable the system to remember rarely occurring stimulus-response events. Reward value based learning would trigger plasticity regardless of what was expected, and thus the traces of rare events would disappear entirely, and would furthermore overlearn frequent associations. Interestingly, with the use of the RPE, traces of events could only disappear if many remappings of unrelated states and actions occurred. Moreover, RPE also implied a reduced energy requirement since modifications of the synaptic weights do not occur all the time, as is the case when the plasticity depends only on the reward value.

### Implementation of the RP pathway

With regard to the RP pathway and considering its implementation, plasticity could occur between RP neurons and dopaminergic neurons in the model. Additionally, the global activity in RP, and not the one of specific state-action coding striosomal sub-population, could in principle code for the RPE. The information regarding current state and selected action would be provided by the active input populations, and synaptic plasticity of these connections would allow learning of the reward prediction for different combinations of states and actions. This would place some constraints on ensuring that neurons in RP fire because the weights of both incoming connections would be plastic. It is also possible that the prediction relies only on the state information, at least in a first phase, and could be refined once the action is considered or selected.

We see three different interdependent design options that can support both experimental evidence and the functional requirements stressed by our model as to how the RP pathway could be set. There is no indication that D1 and D2 receptors would be specific to matrisomes (Friedman et al., 2015; Fujiyama et al., 2015). However, only striosomes sends connections to SNc (Lévesque and Parent, 2005; Amemori et al., 2011; Fujiyama et al., 2011). Furthermore, some striosomal MSNs project to GPi/SNr (Crittenden and Graybiel, 2011). Therefore, assuming an antagonistic role for the D1 and D2 striosomes, a first option could be that an additional pathway, within RP, could code specifically for negative reward or pain. Such a pathway could go via GPi or SNr onto SNc (Fujiyama et al., 2011), essentially constituting an indirect striosomo-nigral pathway originating in striosomal D2 MSNs. This could represent the biological substrate of the negative RP weights. Inputs from LH or periaqueductal gray have also been shown to be critical in negative learning and in pain learning, respectively, indicating they could be involved in coding negative reward prediction (Matsumoto and Hikosaka, 2007; Bromberg-Martin et al., 2010; Roy et al., 2014).

Secondly, we had suggested that projections from striosomes could convey information about the expected reward value to the selection process (Berthet et al., 2012). This information might be valuable when comparing options associated with various expected reward values or probabilities. It would be similar to an involvement of the critic during action selection in the Actor-Critic framework. Commonly associated with this representation is a ventro-dorsal distinction of the striatum (O'Doherty et al., 2004; Voorn et al., 2004; Atallah et al., 2007; Humphries and Prescott, 2010). We suggest a unification of the ventro-dorsal and striosomo-matrisomal distinction. Matrisomes would be involved in action selection based on sensori-motor information and would code action values. Dorsal striosomes would also participate in the selection by supplying information about the expected outcome. Finally, ventral striosomes would be engaged in coding the expected reward as part of the RPE computation and would not be directly engaged in the selection.

A third possibility is that the joint state-action reward prediction, here coded by the striosomes, could be obtained through this described more complex network. Striosomes would thus code only for a state related reward prediction. Knowledge about the action would be received by the dopaminergic neurons from GPi and SNr, a circuit that has been described in biology but the function of which remains unknown (Joel and Weiner, 2000; Crittenden and Graybiel, 2011). The integration of state and action information would therefore take place directly within the dopaminergic nuclei (Cohen et al., 2012).

A caveat to our model is its lack of external expectation, or drive, onto the RP pathway. This would enable the system to escape situations where it expects a bad outcome, and as it eventually obtains it, not to change anything since the RPE would be zero. The model requires a mechanism, e.g., a drive that would set superordinate goals, which would prevent it constantly pushing the system to avoid settling for nothing.

### Implications for Parkinson's disease

The PD simulations did not show the kind of catastrophic performance present in the noD2 condition, even though it was the D2 pathway that was most involved. Our results indicate a differential involvement of the D1 and D2 pathways in PD associated dysfunctions. Furthermore, our model suggests that restoring the ability of cortico-striatal connections onto D1 MSNs to be plastic in Parkinsonian patients might prove to significantly increase learning and action selection performance. In patients, symptoms are usually not observed before the degeneration of a large part of the dopaminergic population (Whone et al., 2003). We see three reasons that could explain the relatively early occurrence of trouble in action selection of our model. First, some homeostatic processes could be involved, delaying the onset of symptoms. Secondly, even though we used a filtered trace of the dopaminergic neurons activity, a small variation from baseline triggered some plasticity in the relevant pathways. We suggest that there might be some thresholding of the dopamine level, preventing minor fluctuations of the weights. Thirdly, our model does not differentiate between VTA and SNc dopaminergic neurons and the simulated cell degeneration causes the same change in baseline dopamine level for all the pathways. However, in PD, the loss of neurons occurs mostly in SNc, which projects mainly to the dorsal striatum whereas VTA projects to the ventral striatum (DeLong, 1990; Alberico et al., 2015). It is therefore possible that the RP system might be less affected than the D1 and D2 pathways during PD.

With respect to the over-sensitivity observed in D2 MSNs in PD patients (Bamford et al., 2004), our model shows that removing the irrelevant input from the D1 pathway in the two most severe PD conditions could drive performance up to the level of the noD1 condition. This condition has a better average success ratio than the two PD conditions at the end of a block. Interestingly, the quantity of D1 receptors is believed to decrease in PD, but the remaining ones are thought to become hypersensitive. This could be the reason for the dyskinesia observed in PD patients treated with L-dopa (Gerfen, 2003). This drug, which alleviates motor symptoms in PD, could rescue the D1 MSN dynamics by raising the tonic level of dopamine, thereby unlocking the D1 weights by restoring the ability of RPE to become positive again.

Additionally, it has been reported that PD patients exhibit better learning from negative than from positive outcomes. This could boil down to the fact that only the D2 pathway can have a beneficial impact on selection because of low dopamine levels. Therefore, it is more valuable since it is impacted by negative RPE (Frank et al., 2004; Cox et al., 2015). Our model supports observations indicating that dopaminergic medication in mild PD patients impaired reversal learning when reversals were signaled by unexpected punishment (Swainson et al., 2000), and that dopamine level in striatum could predict a more pronounced sensibility toward either unexpected rewards or unexpected punishments in a similar task (Cools et al., 2006, 2009). Phasic dopamine dips, coding for a negative RPE and critical for learning the suppression of the selection of an action, are vulnerable to excessive dopamine levels resulting from dopaminergic medication (Frank, 2005). Moreover, for the PD16 and PD33 conditions, an increase of the number of trials within a block proved to be beneficial, suggesting that learning is still possible but hindered by a low learning rate, which seems to be supported by experimental data (Shohamy et al., 2008; Peterson et al., 2009). Even though PD33 did not show a significant difference in performance between the first and the last trials of a block of 40 trials, it did improve when the total number of trials in a block was set to 80. This suggests that the slow change of the D1 weights requires a lot more trials for the population coding for the correct action to overcome the lateral inhibition it receives from the dominant D1 action coding population.

A condition with relatively mild dopaminergic neuron loss might fail to be noticed without a rigorous examination. Results from our model in a condition with low dopaminergic neuron loss (see the PD16 results in Figure 5) suggest that even though the performance during learning are similar to the intact model for the first part of a block, they then fail to improve as much during the second phase.

There is an absence of consensus on the role of the D1 MSNs in PD and on the modifications they may undergo. We suggest that PD could affect D1 MSN dynamics, but since the contribution of D1 in the selection seems to be much smaller relative to D2, the effect might be difficult to detect experimentally or clinically.

### Action selection implementation

Concerning action selection, we assumed that it is actually done at the level of GPi/SNr but obviously depends on the activity in D1 and D2 MSNs (Lo and Wang, 2006). There is also a need to integrate the contributions from a habit learning pathway (Jog et al., 1999; Yin and Knowlton, 2006). Studies on the activation of the D1 and D2 pathways in rat BG seem to support the idea that action selection occurs at this level. Indeed, an increase in the activity of striatal neurons in both the D1 and D2 pathways has been observed during spontaneous movements in mice (Tecuapetla et al., 2014). This indicates the absence of a strict winner-take-all process at this early stage. Additionally, an activity related to a stop signal in SNr has been reported only in the case of successful cancelation (Schmidt et al., 2013).

It has been suggested that exploratory behaviors could be the consequence of a non-uniform initialization of the action values and their expected reward (Kakade and Dayan, 2002). As this unexpected activity is observed for new states, it could offer a way to enforce exploration by biasing it with a negative reward prediction, thus increasing the firing of the dopaminergic neurons. In the tests used here, the diversity of situations does not allow us to assess such hypotheses, but we have nonetheless drawn the initial values of the D1, D2, and RP pathways from a Gaussian distribution. We suggest that the gain of different connections or processes, such as the softmax selection, might rely on neuromodulators such as acetylcholine (Threlfell and Cragg, 2011; Cachope and Cheer, 2014; Nelson et al., 2014), serotonin or noradrenaline. This would imply even a five factor-learning rule: pre- and post-synaptic activity, neuro-modulator A (dopamine), neuro-modulator B, and receptor type. Noradrenaline has been suggested to be involved in modifying the exploration—exploitation ratio of the selection process, in agreement with a gain regulation of our softmax operation (Doya, 2002).

Besides this, an absence of lateral inhibition between matrisomes coding for different actions could be inconvenient if the reaction time depends on the contrast in activity between D1 and D2 MSNs (Lo and Wang, 2006; Collins and Frank, 2014; Bahuguna et al., 2015). Additionally, the increased mean firing rate in this condition to a level well-above what has been observed in biology, further discredits its relevance. We believe that the low number of states and actions along with the simple test setup might be particularly well-suited for the condition without lateral inhibition, but such a condition would fail to perform as well in more complex situations.

## Conclusion

Through analysis of the dynamics and performance of the model, primarily the change of the weights during learning in the various pathways, we were able to formulate new hypotheses regarding the function and organization of different BG network components. Notably, we suggest that some PD symptoms could result from a dysfunctional D1 pathway, whereas the D2 pathway would still be functionally adequate although itself also affected by the reduced dopamine level. Furthermore, we discussed the implementation of the network and detailed the relevant other options, which could be tested experimentally with a specific focus on the integration of the state and action information in BG and with the localization of plasticity in the RP pathway.

## Author contributions

Conceived and designed the experiments: PB, ML, PT, JH, AL. Performed the experiments: PB. Analyzed the data: PB. Writing of the manuscript: PB, ML, PT, JK, AL. Developed the code for the plasticity module: PB, ML, PT.

### Conflict of interest statement

The authors declare that the research was conducted in the absence of any commercial or financial relationships that could be construed as a potential conflict of interest.

## Supplementary material

Table S1 **Parameters and their values used in the simulations**.

Table S2 **Source code repositories of the model**: The data can be reproduced by running the simulation code and module, available as github repositories BCPNN module: https://github.com/pierreberthet/nest-modules BG model: https://github.com/pierreberthet/bg_dopa_nest Additionally, the neural network simulator NEST 2.2.2 (http://www.nest-simulator.org/) and python are required.

## References

[B1] Alberico S. L. Cassell M. D. Narayanan N. S. (2015). The vulnerable ventral tegmental area in Parkinson's disease. Basal Ganglia 5, 51–55. 10.1016/j.baga.2015.06.001 26251824 PMC4523275

[B2] Alexander G. DeLong M. Strick P. L. (1986). Parallel organization of functionally segregated circuits linking basal ganglia and cortex. Annu. Rev. Neurosci. 9, 357–381. 10.1146/annurev.ne.09.030186.002041 3085570

[B3] Amemori K.-I. Gibb L. G. Graybiel A. M. (2011). Shifting responsibly: the importance of striatal modularity to reinforcement learning in uncertain environments. Front. Hum. Neurosci. 5:47. 10.3389/fnhum.2011.00047 21660099 PMC3105240

[B4] Atallah H. E. Lopez-Paniagua D. Rudy J. W. O'Reilly R. C. Reilly R. C. O. (2007). Separate neural substrates for skill learning and performance in the ventral and dorsal striatum. Nat. Neurosci. 10, 126–131. 10.1038/nn1817 17187065

[B5] Atherton J. F. Bevan M. D. (2005). Ionic mechanisms underlying autonomous action potential generation in the somata and dendrites of GABAergic substantia nigra pars reticulata neurons *in vitro*. J. Neurosci. 25, 8272–8281. 10.1523/JNEUROSCI.1475-05.2005 16148235 PMC6725542

[B6] Bahuguna J. Aertsen A. Kumar A. (2015). Existence and Control of Go/No-Go Decision Transition Threshold in the Striatum. PLoS Comput. Biol. 11:e1004233. 10.1371/journal.pcbi.1004233 25910230 PMC4409064

[B7] Bamford N. S. Robinson S. Palmiter R. D. Joyce J. A. Moore C. Meshul C. K. (2004). Dopamine modulates release from corticostriatal terminals. J. Neurosci. 24, 9541–9552. 10.1523/JNEUROSCI.2891-04.2004 15509741 PMC6730145

[B8] Bar-Gad I. Morris G. Bergman H. (2003). Information processing, dimensionality reduction and reinforcement learning in the basal ganglia. Prog Neurobiol. 71, 439–473. 10.1016/j.pneurobio.2003.12.001 15013228

[B9] Bate A. Lindquist M. Edwards I. R. Olsson S. Orre R. Lansner A.. (1998). A Bayesian neural network method for adverse drug reaction signal generation. Eur. J. Clin. Pharmacol. 54, 315–321. 10.1007/s002280050466 9696956

[B10] Bayer H. M. Glimcher P. W. (2005). Midbrain dopamine neurons encode a quantitative reward prediction error signal. Neuron 47, 129–141. 10.1016/j.neuron.2005.05.020 15996553 PMC1564381

[B11] Bell C. C. Han V. Z. Sugawara Y. Grant K. (1997). Synaptic plasticity in a cerebellum-like structure depends on temporal order. Nature 387, 278–281. 10.1038/387278a0 9153391

[B12] Berns G. S. McClure S. M. Pagnoni G. Montague P. R. (2001). Predictability modulates human brain response to reward. J. Neurosci. 21, 2793–2798. 11306631 10.1523/JNEUROSCI.21-08-02793.2001 PMC6762527

[B13] Berretta N. Nisticò R. Bernardi G. Mercuri N. B. (2008). Synaptic plasticity in the basal ganglia: a similar code for physiological and pathological conditions. Prog Neurobiol. 84, 343–362. 10.1016/j.pneurobio.2007.12.004 18243479

[B14] Berthet P. Hellgren-Kotaleski J. Lansner A. (2012). Action selection performance of a reconfigurable basal ganglia inspired model with Hebbian–Bayesian Go-NoGo connectivity. Front. Behav. Neurosci. 6:65. 10.3389/fnbeh.2012.00065 23060764 PMC3462417

[B15] Berthet P. Lansner A. (2014). optogenetic stimulation in a computational model of the basal ganglia biases action selection and reward prediction error. PLoS ONE 9:e90578. 10.1371/journal.pone.0090578 24614169 PMC3948624

[B16] Bi G. Q. Poo M. M. (1998). Synaptic modifications in cultured hippocampal neurons: dependence on spike timing, synaptic strength, and postsynaptic cell type. J. Neurosci. 18, 10464–10472. 9852584 10.1523/JNEUROSCI.18-24-10464.1998 PMC6793365

[B17] Boerlin M. Machens C. K. Denève S. (2013). Predictive Coding of Dynamical Variables in Balanced Spiking Networks. PLoS Comput. Biol. 9:e1003258. 10.1371/journal.pcbi.1003258 24244113 PMC3828152

[B18] Bonci A. Malenka R. C. (1999). Properties and plasticity of excitatory synapses on dopaminergic and GABAergic cells in the ventral tegmental area. J. Neurosci. 19, 3723–3730. 10234004 10.1523/JNEUROSCI.19-10-03723.1999 PMC6782740

[B19] Bromberg-Martin E. S. Matsumoto M. Hong S. Hikosaka O. (2010). A pallidus-habenula-dopamine pathway signals inferred stimulus values. J. Neurophysiol. 104, 1068–1076. 10.1152/jn.00158.2010 20538770 PMC2934919

[B20] Brown J. Bullock D. Grossberg S. (1999). How the basal ganglia use parallel excitatory and inhibitory learning pathways to selectively respond to unexpected rewarding cues. J. Neurosci. 19, 10502–11. 10575046 10.1523/JNEUROSCI.19-23-10502.1999 PMC6782432

[B21] Buesing L. Bill J. Nessler B. Maass W. (2011). Neural dynamics as sampling: a model for stochastic computation in recurrent networks of spiking neurons. PLoS Comput. Biol. 7:e1002211. 10.1371/journal.pcbi.1002211 22096452 PMC3207943

[B22] Cachope R. Cheer J. F. (2014). Local control of striatal dopamine release. Front. Behav. Neurosci. 8:188. 10.3389/fnbeh.2014.00188 24904339 PMC4033078

[B23] Calabresi P. Centonze D. Gubellini P. Marfia G. A. Pisani A. Sancesario G.. (2000). Synaptic transmission in the striatum: from plasticity to neurodegeneration. Prog. Neurobiol. 61, 231–265. 10.1016/S0301-0082(99)00030-1 10727775

[B24] Cardinal R. N. (2006). Neural systems implicated in delayed and probabilistic reinforcement. Neural Netw. 19, 1277–1301. 10.1016/j.neunet.2006.03.004 16938431

[B25] Cohen J. Y. Haesler S. Vong L. Lowell B. B. Uchida N. (2012). Neuron-type-specific signals for reward and punishment in the ventral tegmental area. Nature 482, 85–88. 10.1038/nature10754 22258508 PMC3271183

[B26] Cohen M. X. Frank M. J. (2009). Neurocomputational models of basal ganglia function in learning, memory and choice. Behav. Brain Res. 199, 141–156. 10.1016/j.bbr.2008.09.029 18950662 PMC2762323

[B27] Collins A. G. E. Frank M. J. (2014). Opponent actor learning (OpAL): modeling interactive effects of striatal dopamine on reinforcement learning and choice incentive. Psychol. Rev. 121, 337–366. 10.1037/a0037015 25090423

[B28] Cools R. Altamirano L. D'Esposito M. (2006). Reversal learning in Parkinson's disease depends on medication status and outcome valence. Neuropsychologia 44, 1663–1673. 10.1016/j.neuropsychologia.2006.03.030 16730032

[B29] Cools R. Frank M. J. Gibbs S. E. Miyakawa A. Jagust W. D'Esposito M. (2009). Striatal dopamine predicts outcome-specific reversal learning and its sensitivity to dopaminergic drug administration. J. Neurosci. 29, 1538–1543. 10.1523/JNEUROSCI.4467-08.2009 19193900 PMC2940719

[B30] Cox S. M. L. Frank M. J. Larcher K. Fellows L. K. Clark C. A. Leyton M.. (2015). Striatal D1 and D2 signaling differentially predict learning from positive and negative outcomes. Neuroimage 109, 95–101. 10.1016/j.neuroimage.2014.12.070 25562824

[B31] Crittenden J. R. Graybiel A. M. (2011). Basal ganglia disorders associated with imbalances in the striatal striosome and matrix compartments. Front. Neuroanat. 5:59. 10.3389/fnana.2011.00059 21941467 PMC3171104

[B32] Cui G. Jun S. B. Jin X. Pham M. D. Vogel S. S. Lovinger D. M.. (2013). Concurrent activation of striatal direct and indirect pathways during action initiation. Nature 494, 238–242. 10.1038/nature11846 23354054 PMC4039389

[B33] Daw N. D. Courville A. C. Touretzky D. S. Tourtezky D. S. (2006). Representation and timing in theories of the dopamine system. Neural Comput. 18, 1637–1677. 10.1162/neco.2006.18.7.1637 16764517

[B34] Daw N. D. Doya K. (2006). The computational neurobiology of learning and reward. Curr. Opin. Neurobiol. 16, 199–204. 10.1016/j.conb.2006.03.006 16563737

[B35] DeLong M. R. (1990). Primate models of movement disorders of basal ganglia origin. Trends Neurosci. 13, 281–285. 10.1016/0166-2236(90)90110-V 1695404

[B36] Delong M. R. Georgopoulos A. P. Crutcher M. D. Mitchell S. J. Richardson R. T. Alexander G. E. (1984). Functional organization of the basal ganglia: contributions of single-cell recording studies, in Ciba Foundation Symposium 107 - Functions of the Basal Ganglia, eds Evered D. O'Connor M. (Chichester, UK: John Wiley & Sons, Ltd.), 64–82. 10.1002/9780470720882.ch5 6389041

[B37] Deneve S. (2008). Bayesian spiking neurons I: inference. Neural Comput. 20, 91–117. 10.1162/neco.2008.20.1.91 18045002

[B38] Doig N. M. Moss J. Bolam J. P. (2010). Cortical and thalamic innervation of direct and indirect pathway medium-sized spiny neurons in mouse striatum. J. Neurosci. 30, 14610–14618. 10.1523/JNEUROSCI.1623-10.2010 21048118 PMC6633626

[B39] Doya K. (2002). Metalearning and neuromodulation. Neural Netw. 15, 495–506. 10.1016/S0893-6080(02)00044-8 12371507

[B40] Doya K. (2007). Reinforcement learning: computational theory and biological mechanisms. HFSP J. 1, 30. 10.2976/1.2732246/10.2976/1 19404458 PMC2645553

[B41] Doya K. Ishii S. Pouget A. Rao R. P. N. (2007). Bayesian Brain. Cambridge, MA; London, UK: MIT Press.

[B42] Eblen F. Graybiel A. M. (1995). Highly restricted origin of prefrontal cortical inputs to striosomes in the macaque monkey. J. Neurosci. 15, 5999–6013. 7666184 10.1523/JNEUROSCI.15-09-05999.1995 PMC6577677

[B43] Eppler J. M. Helias M. Muller E. Diesmann M. Gewaltig M.-O. (2008). PyNEST: a convenient interface to the NEST simulator. Front. Neuroinform. 2:12. 10.3389/neuro.11.012.2008 19198667 PMC2636900

[B44] Farries M. A. Fairhall A. L. (2007). Reinforcement learning with modulated spike timing dependent synaptic plasticity. J. Neurophysiol. 98, 3648–3665. 10.1152/jn.00364.2007 17928565

[B45] Fee M. S. (2014). The role of efference copy in striatal learning. Curr. Opin. Neurobiol. 25, 194–200. 10.1016/j.conb.2014.01.012 24566242 PMC4153469

[B46] Fiebig F. Lansner A. (2014). Memory consolidation from seconds to weeks: a three-stage neural network model with autonomous reinstatement dynamics. Front. Comput. Neurosci. 8:64. 10.3389/fncom.2014.00064 25071536 PMC4077014

[B47] Flaherty W. J. Graybiell A. M. (1993). Two input systems for body representations in the primate striatal matrix: experimental evidence in the squirrel monkey. J. Neurosci. 13, 1120–1137. 7680067 10.1523/JNEUROSCI.13-03-01120.1993 PMC6576612

[B48] Frank M. J. (2005). Dynamic dopamine modulation in the basal ganglia: a neurocomputational account of cognitive deficits in medicated and nonmedicated Parkinsonism. J. Cogn. Neurosci. 17, 51–72. 10.1162/0898929052880093 15701239

[B49] Frank M. J. (2006). Hold your horses: a dynamic computational role for the subthalamic nucleus in decision making. Neural Netw. 19, 1120–1136. 10.1016/j.neunet.2006.03.006 16945502

[B50] Frank M. J. Seeberger L. C. O'reilly R. C. (2004). By carrot or by stick: cognitive reinforcement learning in parkinsonism. Science 306, 1940–1943. 10.1126/science.1102941 15528409

[B51] Freeze B. S. Kravitz A. V. Hammack N. Berke J. D. Kreitzer A. C. (2013). Control of basal ganglia output by direct and indirect pathway projection neurons. J. Neurosci. 33, 18531–18539. 10.1523/JNEUROSCI.1278-13.2013 24259575 PMC3834057

[B52] Frémaux N. Sprekeler H. Gerstner W. (2010). Functional requirements for reward-modulated spike-timing-dependent plasticity. J. Neurosci. 30, 13326–13337. 10.1523/JNEUROSCI.6249-09.2010 20926659 PMC6634722

[B53] Frémaux N. Sprekeler H. Gerstner W. (2013). Reinforcement learning using a continuous time actor-critic framework with spiking neurons. PLoS Comput. Biol. 9:e1003024. 10.1371/journal.pcbi.1003024 23592970 PMC3623741

[B54] Friedman A. Homma D. Gibb L. G. G. Amemori K. Rubin S. J. J. Hood A. S. S.. (2015). A corticostriatal path targeting striosomes controls decision-making under conflict. Cell 161, 1320–1333. 10.1016/j.cell.2015.04.049 26027737 PMC4477966

[B55] Fujiyama F. Sohn J. Nakano T. Furuta T. Nakamura K. C. Matsuda W.. (2011). Exclusive and common targets of neostriatofugal projections of rat striosome neurons: a single neuron-tracing study using a viral vector. Eur. J. Neurosci. 33, 668–677. 10.1111/j.1460-9568.2010.07564.x 21314848

[B56] Fujiyama F. Takahashi S. Karube F. Pammi V. S. C. (2015). Morphological elucidation of basal ganglia circuits contributing reward prediction. Front. Neurosci. 9:6. 10.3389/fnins.2015.00006 25698913 PMC4318281

[B57] Fukunaga K. Stoppini L. Miyamoto E. Muller D. (1993). Long-term potentiation is associated with an increased activity of Ca2+/calmodulin-dependent protein kinase II. J. Biol. Chem. 268, 7863–7867. 8385124

[B58] Gerfen C. R. (1989). The neostriatal mosaic: striatal patch-matrix organization is related to cortical lamination. Science 246, 385–388. 10.1126/science.2799392 2799392

[B59] Gerfen C. R. (1992). The neostriatal mosaic: multiple levels of compartmental organization. Trends Neurosci. 15, 133–139. 10.1016/0166-2236(92)90355-C 1374971

[B60] Gerfen C. R. (2003). D1 dopamine receptor supersensitivity in the dopamine-depleted striatum animal model of Parkinson's disease. Neuroscientist 9, 455–462. 10.1177/1073858403255839 14678578

[B61] Gerfen C. R. Engber T. M. Mahan L. C. Susel Z. V. I. Thomas N. Monsma F. J.. (1990). D1 and D2 dopamine receptor-regulated gene expression of striatonigral and striatopallidal neurons. Science 250, 1429–1432. 10.1126/science.2147780 2147780

[B62] Gerfen C. R. Herkenham M. Thibault J. Biochemistry C. De France C. (1987). The neostriatal dopaminergic mosaic : II. Patch- and matrix-directed mesostriatal dopaminergic and non-dopaminergic systems mesostriatal. J. Neurosci. 7, 3915–3934. 2891799 10.1523/JNEUROSCI.07-12-03915.1987 PMC6569093

[B63] Gernert M. Fedrowitz M. Wlaz P. Löscher W. (2004). Subregional changes in discharge rate, pattern, and drug sensitivity of putative GABAergic nigral neurons in the kindling model of epilepsy. Eur. J. Neurosci. 20, 2377–2386. 10.1111/j.1460-9568.2004.03699.x 15525279

[B64] Gershman S. J. Moustafa A. A. Ludvig E. A. (2014). Time representation in reinforcement learning models of the basal ganglia. Front. Comput. Neurosci. 7:194. 10.3389/fncom.2013.00194 24409138 PMC3885823

[B65] Gewaltig M.-O. Diesmann M. (2007). NEST (NEural Simulation Tool). Scholarpedia 2:1430 10.4249/scholarpedia.1430

[B66] Gillies A. Arbuthnott G. (2000). Computational models of the basal ganglia. Mov. Disord. 15, 762–770. 10.1002/1531-8257(200009)15:5<762::AID-MDS1002>3.0.CO;2-2 11009178

[B67] Gittis A. H. Nelson A. B. Thwin M. T. Palop J. J. Kreitzer A. C. (2010). Distinct roles of GABAergic interneurons in the regulation of striatal output pathways. J. Neurosci. 30, 2223–2234. 10.1523/JNEUROSCI.4870-09.2010 20147549 PMC2836801

[B68] Glimcher P. W. (2011). Understanding dopamine and reinforcement learning: the dopamine reward prediction error hypothesis. Proc. Natl. Acad. Sci. U.S.A. 108(Suppl.), 15647–15654. 10.1073/pnas.1014269108 21389268 PMC3176615

[B69] Graybiel A. M. (1995). Building action repertoires: memory and learning functions of the basal ganglia. Curr. Opin. Neurobiol. 5, 733–741. 10.1016/0959-4388(95)80100-6 8805417

[B70] Graybiel A. M. (2005). The basal ganglia: learning new tricks and loving it. Curr. Opin. Neurobiol. 15, 638–644. 10.1016/j.conb.2005.10.006 16271465

[B71] Graybiel A. M. (2008). Habits, rituals, and the evaluative brain. Annu. Rev. Neurosci. 31, 359–387. 10.1146/annurev.neuro.29.051605.112851 18558860

[B72] Graybiel A. M. Hirsch E. C. Agid Y. A. (1987). Differences in tyrosine hydroxylase-like immunoreactivity characterize the mesostriatal innervation of striosomes and extrastriosomal matrix at maturity. Proc. Natl. Acad. Sci. U.S.A. 84, 303–307. 10.1073/pnas.84.1.303 2879289 PMC304192

[B73] Groman S. M. Lee B. London E. D. Mandelkern M. A. James A. S. Feiler K.. (2011). Dorsal striatal D2-like receptor availability covaries with sensitivity to positive reinforcement during discrimination learning. J. Neurosci. 31, 7291–7299. 10.1523/JNEUROSCI.0363-11.2011 21593313 PMC3114883

[B74] Gurney K. N. Humphries M. D. Redgrave P. (2015). A new framework for cortico-striatal plasticity: behavioural theory meets *in vitro* data at the reinforcement-action interface. PLoS Biol. 13:e1002034. 10.1371/journal.pbio.1002034 25562526 PMC4285402

[B75] Gurney K. N. Prescott T. T. J. Redgrave P. (2001). A computational model of action selection in the basal ganglia. I. A new functional anatomy. Biol. Cybern. 84, 401–410. 10.1007/PL00007984 11417052

[B76] Hollerman J. R. Schultz W. (1998). Dopamine neurons report an error in the temporal prediction of reward during learning. Nat. Neurosci. 1, 304–309. 10.1038/1124 10195164

[B77] Houk J. C. Adams J. L. Barto A. G. (1995). A model of how the basal ganglia generate and use neural signals that predict reinforcement, in Models of Information Processing in the Basal Ganglia, Vol. 13, eds Houk J. C. Davis J. L. Beiser D. G. (Cambridge, MA; London, UK: MIT Press), 249–270.

[B78] Hull C. Adesnik H. Scanziani M. (2009). Neocortical disynaptic inhibition requires somatodendritic integration in interneurons. J. Neurosci. 29, 8991–8995. 10.1523/JNEUROSCI.5717-08.2009 19605636 PMC2760400

[B79] Humphries M. D. Prescott T. J. (2010). The ventral basal ganglia, a selection mechanism at the crossroads of space, strategy, and reward. Prog Neurobiol. 90, 385–417. 10.1016/j.pneurobio.2009.11.003 19941931

[B80] Ilango A. Kesner A. J. Keller K. L. Stuber G. D. Bonci A. Ikemoto S. (2014). Similar roles of substantia nigra and ventral tegmental dopamine neurons in reward and aversion. J. Neurosci. 34, 817–822. 10.1523/JNEUROSCI.1703-13.2014 24431440 PMC3891961

[B81] Ito M. Doya K. (2009). Validation of decision-making models and analysis of decision variables in the rat basal ganglia. J. Neurosci. 29, 9861–9874. 10.1523/JNEUROSCI.6157-08.2009 19657038 PMC6666589

[B82] Izhikevich E. M. (2007). Solving the distal reward problem through linkage of STDP and dopamine signaling. Cereb. Cortex 17, 2443–2452. 10.1093/cercor/bhl152 17220510

[B83] Jin D. Z. Fujii N. Graybiel A. M. (2009). Neural representation of time in cortico-basal ganglia circuits. Proc. Natl. Acad. Sci. U.S.A. 106, 19156–19161. 10.1073/pnas.0909881106 19850874 PMC2776432

[B84] Jitsev J. Morrison A. Tittgemeyer M. (2012). Learning from positive and negative rewards in a spiking neural network model of basal ganglia, in The 2012 International Joint Conference on Neural Networks (IJCNN) (Brisbane, QLD: IEEE), 1–8.

[B85] Joel D. Niv Y. Ruppin E. (2002). Actor-critic models of the basal ganglia: new anatomical and computational perspectives. Neural Netw. 15, 535–547. 10.1016/S0893-6080(02)00047-3 12371510

[B86] Joel D. Weiner I. (2000). The connections of the dopaminergic system with the striatum in rats and primates: an analysis with respect to the functional and compartmental organization of the striatum. Neuroscience 96, 451–474. 10.1016/S0306-4522(99)00575-8 10717427

[B87] Jog M. S. Kubota Y. Connolly C. I. Hillegaart V. Graybiel A. M. (1999). Building neural representations of habits. Science 286, 1745–1749. 10.1126/science.286.5445.1745 10576743

[B88] Johnston J. G. Gerfen C. R. Haber S. N. van der Kooy D. (1990). Mechanisms of striatal pattern formation: conservation of mammalian compartmentalization. Dev. Brain Res. 57, 93–102. 10.1016/0165-3806(90)90189-6 1965303

[B89] Jones S. Kornblum J. L. Kauer J. A. (2000). Amphetamine blocks long-term synaptic depression in the ventral tegmental area. J. Neurosci. 20, 5575–5580. 10908593 10.1523/JNEUROSCI.20-15-05575.2000 PMC6772550

[B90] Joyce J. N. Sapp D. W. Marshall J. F. (1986). Human striatal dopamine receptors are organized in compartments. Proc. Natl. Acad. Sci. U.S.A. 83, 8002–8006. 10.1073/pnas.83.20.8002 2945207 PMC386853

[B91] Kakade S. Dayan P. (2002). Dopamine: generalization and bonuses. Neural Netw. 15, 549–559. 10.1016/S0893-6080(02)00048-5 12371511

[B92] Kaplan B. A. Lansner A. (2014). A spiking neural network model of self-organized pattern recognition in the early mammalian olfactory system. Front. Neural Circuits 8:5. 10.3389/fncir.2014.00005 24570657 PMC3916767

[B93] Kemp J. M. Powell T. P. S. (1971). The structure of the caudate nucleus of the cat: light and electron microscopy. Philos. Trans. R. Soc. B Biol. Sci. 262, 383–401. 10.1098/rstb.1971.0102 4107495

[B94] Kimura M. Minamimoto T. Matsumoto N. Hori Y. (2004). Monitoring and switching of cortico-basal ganglia loop functions by the thalamo-striatal system. Neurosci. Res. 48, 335–360. 10.1016/j.neures.2003.12.002 15041188

[B95] Kiyatkin E. A. Stein E. A. (1995). Fluctuations in nucleus accumbens dopamine during cocaine self-administration behavior: an *in vivo* electrochemical study. Neuroscience 64, 599–617. 10.1016/0306-4522(94)00436-9 7715774

[B96] Körding K. P. Wolpert D. M. (2004). Bayesian integration in sensorimotor learning. Nature 427, 244–247. 10.1038/nature02169 14724638

[B97] Kravitz A. V. Freeze B. S. Parker P. R. L. Kay K. Thwin M. T. Deisseroth K.. (2010). Regulation of parkinsonian motor behaviours by optogenetic control of basal ganglia circuitry. Nature 466, 622–626. 10.1038/nature09159 20613723 PMC3552484

[B98] Kravitz A. V. Tye L. D. Kreitzer A. C. (2012). Distinct roles for direct and indirect pathway striatal neurons in reinforcement. Nat. Neurosci. 15, 816–818. 10.1038/nn.3100 22544310 PMC3410042

[B99] Kreitzer A. C. Malenka R. C. (2007). Endocannabinoid-mediated rescue of striatal LTD and motor deficits in Parkinson's disease models. Nature 445, 643–647. 10.1038/nature05506 17287809

[B100] Kwak S. Huh N. Seo J.-S. Lee J.-E. Han P.-L. Jung M. W. (2014). Role of dopamine D2 receptors in optimizing choice strategy in a dynamic and uncertain environment. Front. Behav. Neurosci. 8:368. 10.3389/fnbeh.2014.00368 25389395 PMC4211411

[B101] Lavin A. Nogueira L. Lapish C. C. Wightman R. M. Phillips P. E. M. Seamans J. K. (2005). Mesocortical dopamine neurons operate in distinct temporal domains using multimodal signaling. J. Neurosci. 25, 5013–5023. 10.1523/JNEUROSCI.0557-05.2005 15901782 PMC5509062

[B102] Legenstein R. Pecevski D. Maass W. (2008). A learning theory for reward-modulated spike-timing-dependent plasticity with application to biofeedback. PLoS Comput. Biol. 4:e1000180. 10.1371/journal.pcbi.1000180 18846203 PMC2543108

[B103] Lévesque M. Parent A. (2005). The striatofugal fiber system in primates: a reevaluation of its organization based on single-axon tracing studies. Proc. Natl. Acad. Sci. U.S.A. 102, 11888–11893. 10.1073/pnas.0502710102 16087877 PMC1187973

[B104] Limousin P. Pollak P. Benazzouz A. Hoffmann D. Le Bas J.-F. Broussolle E.. (1995). Effect on parkinsonian signs and subthalamic nucleus stimulation symptoms of bilateral. Lancet 345, 91–95. 10.1016/S0140-6736(95)90062-4 7815888

[B105] Lindahl M. Kamali Sarvestani I. Ekeberg O. Kotaleski J. H. (2013). Signal enhancement in the output stage of the basal ganglia by synaptic short-term plasticity in the direct, indirect, and hyperdirect pathways. Front. Comput. Neurosci. 7:76. 10.3389/fncom.2013.00076 23801960 PMC3685803

[B106] Lisman J. (2014). Two-phase model of the basal ganglia: implications for discontinuous control of the motor system. Philos. Trans. R. Soc. B Biol. Sci. 369:20130489. 10.1098/rstb.2013.0489 25267829 PMC4186240

[B107] Lo C.-C. Wang X.-J. (2006). Cortico-basal ganglia circuit mechanism for a decision threshold in reaction time tasks. Nat. Neurosci. 9, 956–963. 10.1038/nn1722 16767089

[B108] Lundqvist M. Herman P. Lansner A. (2011). Theta and gamma power increases and alpha/beta power decreases with memory load in an attractor network model. J. Cogn. Neurosci. 23, 3008–3020. 10.1162/jocn_a_00029 21452933

[B109] Lüscher C. Malenka R. C. (2011). Drug-evoked synaptic plasticity in addiction: from molecular changes to circuit remodeling. Neuron 69, 650–663. 10.1016/j.neuron.2011.01.017 21338877 PMC4046255

[B110] Markram H. Lübke J. Frotscher M. Sakmann B. (1997). Regulation of synaptic efficacy by coincidence of postsynaptic APs and EPSPs. Science 275, 213–215. 10.1126/science.275.5297.213 8985014

[B111] Marsden C. D. Obeso J. A. (1994). The functions of the basal ganglia and the paradox of stereotaxic surgery in Parkinson's disease. Brain 117, 877–897. 10.1093/brain/117.4.877 7922472

[B112] Matsuda W. Furuta T. Nakamura K. C. Hioki H. Fujiyama F. Arai R.. (2009). Single nigrostriatal dopaminergic neurons form widely spread and highly dense axonal arborizations in the neostriatum. J. Neurosci. 29, 444–453. 10.1523/JNEUROSCI.4029-08.2009 19144844 PMC6664950

[B113] Matsumoto M. Hikosaka O. (2007). Lateral habenula as a source of negative reward signals in dopamine neurons. Nature 447, 1111–1115. 10.1038/nature05860 17522629

[B114] McGeorge A. J. Faull R. L. M. (1989). The organization of the projection from the cerebral cortex to the striatum in the rat. Neuroscience 29, 503–537. 10.1016/0306-4522(89)90128-0 2472578

[B115] McHaffie J. G. Stanford T. R. Stein B. E. Coizet V. Redgrave P. (2005). Subcortical loops through the basal ganglia. Trends Neurosci. 28, 401–407. 10.1016/j.tins.2005.06.006 15982753

[B116] Meffin H. Burkitt A. N. Grayden D. B. (2004). An analytical model for the “large, fluctuating synaptic conductance state” typical of neocortical neurons *in vivo*. J. Comput. Neurosci. 16, 159–175. 10.1023/B:JCNS.0000014108.03012.81 14758064

[B117] Meli C. Lansner A. (2013). A modular attractor associative memory with patchy connectivity and weight pruning. Network 24, 129–150. 10.3109/0954898X.2013.859323 24251411

[B118] Mengual E. de las Heras S. Erro E. Lanciego J. L. Giménez-Amaya J. M. (1999). Thalamic interaction between the input and the output systems of the basal ganglia. J. Chem. Neuroanat. 16, 187–200. 10.1016/S0891-0618(99)00010-1 10422738

[B119] Merchán-Pérez A. Rodriguez J.-R. Ribak C. E. DeFelipe J. (2009). Proximity of excitatory and inhibitory axon terminals adjacent to pyramidal cell bodies provides a putative basis for nonsynaptic interactions. Proc. Natl. Acad. Sci. U.S.A. 106, 9878–9883. 10.1073/pnas.0900330106 19487685 PMC2701041

[B120] Mink J. W. (1996). The basal ganglia: focused selection and inhibition of competing motor programs. Prog. Neurobiol. 50, 381–425. 10.1016/S0301-0082(96)00042-1 9004351

[B121] Morita K. Morishima M. Sakai K. Kawaguchi Y. (2012). Reinforcement learning: computing the temporal difference of values via distinct corticostriatal pathways. Trends Neurosci. 35, 457–467. 10.1016/j.tins.2012.04.009 22658226

[B122] Nair A. G. Gutierrez-Arenas O. Eriksson O. Vincent P. Hellgren-Kotaleski J. (2015). Sensing positive versus negative reward signals through adenylyl cyclase coupled GPCRs in direct and indirect pathway striatal medium spiny neurons. J. Neurosci. 35, 14017–14030. 10.1523/jneurosci.0730-15.2015 26468202 PMC4604235

[B123] Nakamura K. C. Fujiyama F. Furuta T. Hioki H. Kaneko T. (2009). Afferent islands are larger than mu-opioid receptor patch in striatum of rat pups. Neuroreport 20, 584–588. 10.1097/WNR.0b013e328329cbf9 19287319

[B124] Nambu A. (2008). Seven problems on the basal ganglia. Curr. Opin. Neurobiol. 18, 595–604. 10.1016/j.conb.2008.11.001 19081243

[B125] Nelson A. B. Hammack N. Yang C. F. Shah N. M. Seal R. P. Kreitzer A. C. (2014). Striatal cholinergic interneurons drive GABA release from dopamine terminals. Neuron 82, 63–70. 10.1016/j.neuron.2014.01.023 24613418 PMC3976769

[B126] O'Doherty J. Dayan P. Schultz J. Deichmann R. Friston K. Dolan R. J. (2004). Dissociable roles of ventral and dorsal striatum in instrumental conditioning. Science 304, 452–454. 10.1126/science.1094285 15087550

[B127] O'Reilly R. C. Frank M. J. (2006). Making working memory work: a computational model of learning in the prefrontal cortex and basal ganglia. Neural Comput. 18, 283–328. 10.1162/089976606775093909 16378516

[B128] Obeso J. A. Rodriguez-Oroz M. C. Rodriguez M. Lanciego J. L. Artieda J. Gonzalo N.. (2000). Pathophysiology of the basal ganglia in Parkinson's disease. Trends Neurosci. 23, S8–S19. 10.1016/s1471-1931(00)00028-8 11052215

[B129] Pagnoni G. Zink C. F. Montague P. R. Berns G. S. (2002). Activity in human ventral striatum locked to errors of reward prediction. Nat. Neurosci. 5, 97–98. 10.1038/nn802 11802175

[B130] Paille V. Fino E. Du K. Morera-Herreras T. Perez S. Kotaleski J. H.. (2013). GABAergic circuits control spike-timing-dependent plasticity. J. Neurosci. 33, 9353–9363. 10.1523/JNEUROSCI.5796-12.2013 23719804 PMC6618570

[B131] Parent A. (1990). Extrinsic connections of the basal ganglia. Trends Neurosci. 13, 254–258. 10.1016/0166-2236(90)90105-J 1695399

[B132] Parent A. Hazrati L. N. (1995). Functional anatomy of the basal ganglia. I. The cortico-basal ganglia-thalamo-cortical loop. Brain Res. Rev. 20, 91–127. 10.1016/0165-0173(94)00007-C 7711769

[B133] Pawlak V. Kerr J. N. D. (2008). Dopamine receptor activation is required for corticostriatal spike-timing-dependent plasticity. J. Neurosci. 28, 2435–2446. 10.1523/JNEUROSCI.4402-07.2008 18322089 PMC6671189

[B134] Pawlak V. Wickens J. R. Kirkwood A. Kerr J. N. D. (2010). Timing is not everything: neuromodulation opens the STDP gate. Front. Synaptic Neurosci. 2:146. 10.3389/fnsyn.2010.00146 21423532 PMC3059689

[B135] Peterson D. A. Elliott C. Song D. D. Makeig S. Sejnowski T. J. Poizner H. (2009). Probabilistic reversal learning is impaired in Parkinson's disease. Neuroscience 163, 1092–1101. 10.1016/j.neuroscience.2009.07.033 19628022 PMC2760640

[B136] Pfister J.-P. Gerstner W. (2006). Triplets of spikes in a model of spike timing-dependent plasticity. J. Neurosci. 26, 9673–9682. 10.1523/JNEUROSCI.1425-06.2006 16988038 PMC6674434

[B137] Potjans W. Diesmann M. Morrison A. (2011). An imperfect dopaminergic error signal can drive temporal-difference learning. PLoS Comput. Biol. 7:e1001133. 10.1371/journal.pcbi.1001133 21589888 PMC3093351

[B138] Potjans W. Morrison A. Diesmann M. (2009). A spiking neural network model of an actor-critic learning agent. Neural Comput. 21, 301–339. 10.1162/neco.2008.08-07-593 19196231

[B139] Potjans W. Morrison A. Diesmann M. (2010). Enabling functional neural circuit simulations with distributed computing of neuromodulated plasticity. Front. Comput. Neurosci. 4:141. 10.3389/fncom.2010.00141 21151370 PMC2996144

[B140] Rauch A. La Camera G. Luscher H.-R. Senn W. Fusi S. (2003). Neocortical pyramidal cells respond as integrate-and-fire neurons to *in vivo*-like input currents. J. Neurophysiol. 90, 1598–1612. 10.1152/jn.00293.2003 12750422

[B141] Redgrave P. Gurney K. (2006). The short-latency dopamine signal: a role in discovering novel actions? Nat. Rev. Neurosci. 7, 967–975. 10.1038/nrn2022 17115078

[B142] Redgrave P. Prescott T. J. Gurney K. N. (1999). The basal ganglia: a vertebrate solution to the selection problem? Neuroscience 89, 1009–1023. 10.1016/S0306-4522(98)00319-4 10362291

[B143] Ren M. Yoshimura Y. Takada N. Horibe S. Komatsu Y. (2007). Specialized inhibitory synaptic actions between nearby neocortical pyramidal neurons. Science 316, 758–761. 10.1126/science.1135468 17478724

[B144] Reynolds J. N. J. Wickens J. R. (2002). Dopamine-dependent plasticity of corticostriatal synapses. Neural Netw. 15, 507–521. 10.1016/S0893-6080(02)00045-X 12371508

[B145] Reynolds J. Wickens J. (2000). Substantia nigra dopamine regulates synaptic plasticity and membrane potential fluctuations in the rat neostriatum, *in vivo*. Neuroscience 99, 199–203. 10.1016/S0306-4522(00)00273-6 10938425

[B146] Rivest F. Kalaska J. F. Bengio Y. (2010). Alternative time representation in dopamine models. J. Comput. Neurosci. 28, 107–130. 10.1007/s10827-009-0191-1 19847635

[B147] Robinson S. Smith D. M. Mizumori S. J. Y. Palmiter R. D. (2004). Firing properties of dopamine neurons in freely moving dopamine-deficient mice: effects of dopamine receptor activation and anesthesia. Proc. Natl. Acad. Sci. U.S.A. 101, 13329–13334. 10.1073/pnas.0405084101 15317940 PMC516529

[B148] Romanelli P. Esposito V. Schaal D. W. Heit G. (2005). Somatotopy in the basal ganglia: experimental and clinical evidence for segregated sensorimotor channels. Brain Res. Brain Res. Rev. 48, 112–128. 10.1016/j.brainresrev.2004.09.008 15708631

[B149] Roy M. Shohamy D. Daw N. Jepma M. Wimmer G. E. Wager T. D. (2014). Representation of aversive prediction errors in the human periaqueductal gray. Nat. Neurosci. 17, 1607–1612. 10.1038/nn.3832 25282614 PMC4213247

[B150] Ruan H. Saur T. Yao W.-D. (2014). Dopamine-enabled anti-Hebbian timing-dependent plasticity in prefrontal circuitry. Front. Neural Circuits 8:38. 10.3389/fncir.2014.00038 24795571 PMC4005942

[B151] Samejima K. Ueda Y. Doya K. Kimura M. (2005). Representation of action-specific reward values in the striatum. Science 310, 1337–1340. 10.1126/science.1115270 16311337

[B152] Samson R. D. Frank M. J. Fellous J.-M. (2010). Computational models of reinforcement learning: the role of dopamine as a reward signal. Cogn. Neurodyn. 4, 91–105. 10.1007/s11571-010-9109-x 21629583 PMC2866366

[B153] Sandberg A. (2003). Bayesian Attractor Neural Network Models of Memory. Available online at: http://papers://c941067e-36da-4589-acf1-2f5738fdb5a1/Paper/p715

[B154] Sandberg A. Lansner A. Petersson K. M. Ekeberg O. Ekeberg G. (2000). A palimpsest memory based on an incremental Bayesian learning rule. Neurocomputing 32–33, 987–994. 10.1016/S0925-2312(00)00270-8

[B155] Schmidt R. Leventhal D. K. Mallet N. Chen F. Berke J. D. (2013). Canceling actions involves a race between basal ganglia pathways. Nat. Neurosci. 16, 1118–1124. 10.1038/nn.3456 23852117 PMC3733500

[B156] Schroll H. Hamker F. H. (2013). Computational models of basal-ganglia pathway functions: focus on functional neuroanatomy. Front. Syst. Neurosci. 7:122. 10.3389/fnsys.2013.00122 24416002 PMC3874581

[B157] Schultz W. Dayan P. Montague P. R. (1997). A neural substrate of prediction and reward. Science 275, 1593–1599. 10.1126/science.275.5306.1593 9054347

[B158] Sesack S. R. Grace A. A. (2010). Cortico-Basal ganglia reward network: microcircuitry. Neuropsychopharmacology 35, 27–47. 10.1038/npp.2009.93 19675534 PMC2879005

[B159] Shen W. Flajolet M. Greengard P. Surmeier D. J. (2008). Dichotomous dopaminergic control of striatal synaptic plasticity. Science 321, 848–851. 10.1126/science.1160575 18687967 PMC2833421

[B160] Shohamy D. Myers C. E. Kalanithi J. Gluck M. A. (2008). Basal ganglia and dopamine contributions to probabilistic category learning. Neurosci. Biobehav. Rev. 32, 219–236. 10.1016/j.neubiorev.2007.07.008 18061261 PMC2705841

[B161] Stephenson-Jones M. Kardamakis A. A. Robertson B. Grillner S. (2013). Independent circuits in the basal ganglia for the evaluation and selection of actions. Proc. Natl. Acad. Sci. U.S.A. 110, E3670–E3679. 10.1073/pnas.1314815110 24003130 PMC3780871

[B162] Stewart T. C. Bekolay T. Eliasmith C. (2012). Learning to select actions with spiking neurons in the Basal Ganglia. Front. Neurosci. 6:2. 10.3389/fnins.2012.00002 22319465 PMC3269066

[B163] Stocco A. Lebiere C. Anderson J. R. (2010). Conditional routing of information to the cortex: a model of the basal ganglia's role in cognitive coordination. Psychol. Rev. 117, 541–574. 10.1037/a0019077 20438237 PMC3064519

[B164] Suri R. E. (2002). 2002 Special issue TD models of reward predictive responses in dopamine neurons. Neural Netw. 15, 523–533. 10.1016/S0893-6080(02)00046-1 12371509

[B165] Suri R. E. R. Schultz W. (2001). Temporal difference model reproduces anticipatory neural activity. Neural Comput. 862, 841–862. 10.1162/089976601300014376 11255572

[B166] Suri R. E. Schultz W. (1999). A neural network model with dopamine-like reinforcement signal that learns a spatial delayed response task. Neuroscience 91, 871–90. 10.1016/S0306-4522(98)00697-6 10391468

[B167] Surmeier D. J. Ding J. Day M. Wang Z. Shen W. (2007). D1 and D2 dopamine-receptor modulation of striatal glutamatergic signaling in striatal medium spiny neurons. Trends Neurosci. 30, 228–235. 10.1016/j.tins.2007.03.008 17408758

[B168] Sutton R. S. Barto A. G. (1998). Reinforcement Learning. MIT Press Available online at: http://journals.cambridge.org/production/action/cjoGetFulltext?fulltextid=34656 (Accessed April 27, 2012).

[B169] Swainson R. Rogers R. D. Sahakian B. J. Summers B. A. Polkey C. E. Robbins T. W. (2000). Probabilistic learning and reversal deficits in patients with Parkinson's disease or frontal or temporal lobe lesions: possible adverse effects of dopaminergic medication. Neuropsychologia 38, 596–612. 10.1016/S0028-3932(99)00103-7 10689037

[B170] Szydlowski S. N. Pollak Dorocic I. Planert H. Carlén M. Meletis K. Silberberg G. (2013). Target selectivity of feedforward inhibition by striatal fast-spiking interneurons. J. Neurosci. 33, 1678–1683. 10.1523/JNEUROSCI.3572-12.2013 23345240 PMC6618742

[B171] Tai L.-H. Lee A. M. Benavidez N. Bonci A. Wilbrecht L. (2012). Transient stimulation of distinct subpopulations of striatal neurons mimics changes in action value. Nat. Neurosci. 15, 1281–1289. 10.1038/nn.3188 22902719 PMC3951287

[B172] Taverna S. Ilijic E. Surmeier D. J. (2008). Recurrent collateral connections of striatal medium spiny neurons are disrupted in models of Parkinson's disease. J. Neurosci. 28, 5504–5512. 10.1523/JNEUROSCI.5493-07.2008 18495884 PMC3235738

[B173] Tecuapetla F. Matias S. Dugue G. P. Mainen Z. F. Costa R. M. (2014). Balanced activity in basal ganglia projection pathways is critical for contraversive movements. Nat. Commun. 5, 4315. 10.1038/ncomms5315 25002180 PMC4102112

[B174] Tepper J. M. Tecuapetla F. Koós T. Ibáñez-Sandoval O. (2010). Heterogeneity and diversity of striatal GABAergic interneurons. Front. Neuroanat. 4:150. 10.3389/fnana.2010.00150 21228905 PMC3016690

[B175] Threlfell S. Cragg S. J. (2011). Dopamine signaling in dorsal versus ventral striatum: the dynamic role of cholinergic interneurons. Front. Syst. Neurosci. 5:11. 10.3389/fnsys.2011.00011 21427783 PMC3049415

[B176] Tsai H.-C. Zhang F. Adamantidis A. Stuber G. D. Bonci A. de Lecea L.. (2009). Phasic firing in dopaminergic neurons is sufficient for behavioral conditioning. Science 324, 1080–1084. 10.1126/science.1168878 19389999 PMC5262197

[B177] Tully P. J. Hennig M. H. Lansner A. (2014). Synaptic and nonsynaptic plasticity approximating probabilistic inference. Front. Synaptic Neurosci. 6:8. 10.3389/fnsyn.2014.00008 24782758 PMC3986567

[B178] Ungless M. A. Magill P. J. Bolam J. P. (2004). Uniform inhibition of dopamine neurons in the ventral tegmental area by aversive stimuli. Science 303, 2040–2042. 10.1126/science.1093360 15044807

[B179] Voorn P. Vanderschuren L. J. M. Groenewegen H. J. Robbins T. W. Pennartz C. M. (2004). Putting a spin on the dorsal–ventral divide of the striatum. Trends Neurosci. 27, 468–474. 10.1016/j.tins.2004.06.006 15271494

[B180] Whone A. L. Moore R. Y. Piccini P. P. Brooks D. J. (2003). Plasticity of the nigropallidal pathway in Parkinson's disease. Ann. Neurol. 53, 206–213. 10.1002/ana.10427 12557287

[B181] Wickens J. R. Reynolds J. N. J. Hyland B. I. (2003). Neural mechanisms of reward-related motor learning. Curr. Opin. Neurobiol. 13, 685–690. 10.1016/j.conb.2003.10.013 14662369

[B182] Yagishita S. Hayashi-Takagi A. Ellis-Davies G. C. R. Urakubo H. Ishii S. Kasai H. (2014). A critical time window for dopamine actions on the structural plasticity of dendritic spines. Science 345, 1616–1620. 10.1126/science.1255514 25258080 PMC4225776

[B183] Yin H. H. Knowlton B. J. (2006). The role of the basal ganglia in habit formation. Nat. Rev. Neurosci. 7, 464–476. 10.1038/nrn1919 16715055

