# Supplementary material for: Functional Relevance of Different Basal Ganglia Pathways Investigated in a Spiking Model with Reward Dependent Plasticity
Source: Front Neural Circuits. 2016 Jul 21;10:53. doi: 10.3389/fncir.2016.00053 (PMC4954853; doi:10.3389/fncir.2016.00053)
Supplement: Table S1 — Parameters and their values used in the simulations. [file Table1.DOCX]

| 1. MODEL SUMMARY | |
| --- | --- |
| Neuron Model | Leaky IAF |
| Synapse model | Conductance-based with α-shaped PSCs,  Plastic BCPNN synapses with volume transmitter  or static synapses |
| Channel | K^+^ channel (relates to *I_βj_*) |
| Input model | Poisson spike trains |
| Measured quantities | Spike activity, connection strengths, biases |
| State (cortex) layer | 3 states, 60 neurons each |
| Striatum layer:   - Matrisomes - Striosomes | 3 actions:  30 neurons for each actions, for each D1 and D2  15 neurons for each of the 9 state-action pairings |
| GPi/SNr layer | 3 actions, 10 neurons each |
| SNc/VTA | 200 neurons |
| 1. NEURON MODEL | |
| Leaky IAF dynamics | Subthreshold membrane potential *V_m_* of neuron *j* with *n* inputs:  $-C_{m}\frac{{dV}_{m}}{dt}=g_{L}\left( V_{m}-E_{L} \right)+\sum_{i=1}^{n} g_{ex,i}\left( V_{m}-E_{ex,i} \right)+\sum_{i=1}^{n} g_{inh,i}\left( V_{m}-E_{inh,i} \right)+\phi I_{\beta_{j}}$  Spiking: if *V_m_* ≥ *V_th_* spike generated and *V_m_* held at *V_res_* for *t_ref_* ms |
| Parameters of striatal and dopaminergic neurons  Parameters of all other neurons  (NEST 2.2.2 default values) | *C_m_* = 150 ± 5 pF membrane capacitance  *g_L_* = 16.67 nS leak conductance  *E* = -70 mV leak reversal potential  *E_ex_* = 0 mV excitatory reversal potential  *Ei_nh_* = -85 mV inhibitory reversal potential  ϕ = 20 pA current scaling factor  *V_th_* = -45 ± 1 mV membrane voltage threshold  *V_res_* = -80 ± 1 mV membrane reset potential  *t_ref_* = 2 ms refractory period  *dt* = 0.1 ms time resolution  $\Delta t$= 1 ms spike event duration  *C_m_* = 250 ± 5 pF membrane capacitance  *g_L_* = 16.67 nS leak conductance  *E_L_*= -70 mV leak reversal potential  *E_ex_* = 0 mV excitatory reversal potential  *Ei_nh_*= -85 mV inhibitory reversal potential  *V_th_* = -55 ± 1 mV membrane voltage threshold  *V_res_* = -60 ± 1 mV membrane reset potential  *t_ref_* = 2 ms refractory period  *dt* = 0.1 ms time resolution  $\Delta t$ = 1 ms spike event duration |
| 1. CHANNEL MODEL | |
| Activity-dependent hyperpolarizing | K^+^/CAN current of neuron *j*, *Iβ_j_* pA:  $\tau_{p}\frac{dP_{i}}{dt}=\kappa{(E}_{i}-P_{i})$  ${\phi I}_{\beta_{j}}=\phi\beta_{j}=\phi log(P_{j})$  See Equation 4 for calculation of *E_j_* |
| Parameters   - Matrisomes - Dopaminergic neurons | τ*_zj_* = 6 ms *Z_j_* trace time constant  τ*_e_* = 20 ms *E* trace time constant  τ*_p_* = 1000 ms *P* trace time constant  *f_max_* = 40 Hz highest firing rate  $\Delta t$ = 1 ms spike event duration  Same parameters as matrisomes, except:  τ*_zj_* = 15 ms *Z_j_* trace time constant  τ*_p_* = 5000 ms *P* trace time constant  *f_max_* = 20 Hz highest firing rate |
| 1. VOLUME TRANSMITTER | |
| Dopamine level | $\kappa_{D1}=\left\{ \begin{aligned} {(\sigma_{D1}\left( \beta_{dopa}+q \right))}^{\lambda_{D1}}>0 \\ 0 \end{aligned} \right.$  $\kappa_{D2}=\left\{ \begin{aligned} {-(\sigma_{D2}\left( \beta_{dopa}+q \right))}^{\lambda_{D2}}>0 \\ 0 \end{aligned} \right.$  ${\kappa_{RP}=(\sigma_{RP}\left( \beta_{dopa}+q \right))}^{\lambda_{RP}}$ |
| Parameters   - Matrisomes - Dopaminergic neurons | λ*_D1_* = λ*_D2_* = 7  *β_dopa_* = -0.049  *σ_D1_* = *σ_D2_* = 40  τ*_q_* = 100 ms  same parameters as matrisomes, except:  λ*_RP_* = 2  σ*_RP_* = 12 |
| 1. SYNAPSE MODEL | |
| α-shaped PSC dynamics | Excitatory *g_ex_* and inhibitory *g_inh_* conductance changes for postsynaptic neuron *j* with spike at time *t_sp_* by one of the *n* pre-synaptic neurons:  $g_{ex\vert inh,i}\left( t \right)=g_{max}w_{ij}\frac{t-t_{sp}^{i}-d}{\tau_{ex\vert inh}}e^{1-\frac{(t-t_{sp}^{i}-d)}{\tau_{ex\vert inh}}}$ |
| BCPNN synapse | Synaptic strength between *i* and *j*, *w_ij_* nS:  $\tau_{p}\frac{dP_{i}}{dt}=\kappa{(E}_{i}-P_{i}) \tau_{p}\frac{dP_{j}}{dt}=\kappa(E_{j}-P_{j}) \tau_{e}\frac{dP_{ij}}{dt}=\kappa(E_{ij}-P_{ij})$  $w_{ij}=log(\frac{P_{ij}}{P_{i}P_{j}})$  See Equation 4 for calculation of *E_i_*, *E_j_* and *E_ij_*. |
| Parameters   - Matrisomes - Dopaminergic neurons | τ*_zi_* = 5 ms *Z* trace time constant  τ*_e_* = 20 ms *E* trace time constant  τ*_p_* = 1000 ms P trace time constant  *g_max_* = 5 nS peak conductance  τ*_ex_* = 0.2 ms α rise time for excitatory input  τ*_inh_* = 2ms α rise for inhibitory input  *d* = 1 ms transmission delay  same parameters as matrisomes, except:  τ*_zi_* = 12 ms *Z* trace time constant  τ*_p_* = 5000 ms P trace time constant  Additionally, as these connections are set to be inhibitory, activity is fed to the inhibitory channel. |

S1 Table: Parameters and their values used in the simulations.
